# *Kolteria novifilia,* a novel planctomycetotal strain from the volcanic habitat of Panarea divides by unusual lateral budding

**DOI:** 10.1128/jb.00337-24

**Published:** 2025-06-24

**Authors:** Nicolai Kallscheuer, Christian Boedeker, Sandra Wiegand, Timo Kohn, Anja Heuer, Jörg Overmann, Stijn Peters, Mareike Jogler, Manfred Rohde, Christian Jogler

**Affiliations:** 1 Department of Microbial Interactions, Friedrich Schiller-University 9378, Jena, Germany; 2 Leibniz Institute DSMZ-German Collection of Microorganisms and Cell Cultures 28351, Brunswick, Germany; 3 Institute for Biological Interfaces 5, Karlsruhe Institute of Technology 210446 , Eggenstein-Leopoldshafen, Germany; 4 Department of Microbiology, Radboud University 234135, Nijmegen, the Netherlands; 5 Microbiology, Technical University of Braunschweig, Braunschweig, Germany; 6 Central Facility for Microscopy, Helmholtz Centre for Infection Research, Braunschweig, Germany; 7 Cluster of Excellence Balance of the Microverse, Friedrich Schiller University 9378, Jena, Germany; University of Massachusetts Chan Medical School, Worcester, Massachusetts, USA

**Keywords:** planctomycetes, cell division, budding, binary fission, surface layer, *Panarea*, 16S rRNA, V3 region

## Abstract

Members of the phylum *Planctomycetota* are ubiquitous bacteria that play important roles in the global carbon and nitrogen cycle. In this study, we sampled the shallow-sea hydrothermal vent system close to Panarea Island, Italy, and analyzed the bacterial diversity in this habitat using a cultivation-independent amplicon sequencing approach. Motivated by the observed abundance of members of the phylum *Planctomycetota*, we employed cultivation conditions that facilitate the enrichment of planctomycetes and isolated strain Pan216^T^. This strain shows a rarely observed type of cell division—lateral budding. Based on 16S rRNA gene- and multi-locus sequence analyses, its phylogenetic position in the phylum *Planctomycetota* appears vague. Strain Pan216^T^ clustered between the different described families in the class *Planctomycetia*. The novel isolate shares the highest sequence identity (85.4%) of the 16S rRNA gene with *Thermostilla marina* SVX8^T^, indicating that this strain belongs to a novel family. In addition to its uncommon cell division mode, Pan216^T^ cells are pill-shaped and covered by a putative outer surface layer. Genomic analyses of strain Pan216^T^ revealed many giant genes, putative S-layer protein-encoding genes, and only a limited set of canonical cell division genes. Based on the results of the polyphasic analysis, we conclude that strain Pan216^T^ constitutes a novel family within the phylum *Planctomycetota*, for which we propose the name *Kolteriaceae* fam. nov. The novel species *Kolteria novifilia* gen. nov., sp. nov. is represented by the type strain Pan216^T^ (= DSM 100414^T^ = CECT 9536^T^).

**IMPORTANCE:**

We describe a novel family of the underrepresented bacterial phylum *Planctomycetota* that divides by unusual lateral budding. Our strain is the only validly described species that uses this mode of cell division. Furthermore, it represents the only planctomycete outside of the anammox bacteria that has an S-layer-like structure. Taken together, the novel family shows a novel mechanism of cell division that could only be studied in this species.

## INTRODUCTION

The phylum *Planctomycetota* is of ecological importance as its members play key roles in the global carbon and nitrogen cycle (1, 2). The phyla *Verrucomicrobiota*, *Lentisphaerota*, *Kiritimatiellaeota,* “*Candidatus* Omnitrophota,” *Chlamydiota*, and *Planctomycetota* constitute the PVC superphylum (3, 4). Planctomycetes have been isolated from various habitats on Earth in which they occur either free-living or associated with prokaryotes and eukaryotes (5–7). Particularly high abundances of members of the *Planctomycetota—*in the range of 70%–85% of the bacterial community—were observed on the surface of macroscopic phototrophs, for example the kelp *Laminaria hyperborea* or the seagrass *Posidonia oceanica* (6, 8), or in association with metalliferous deposits from hydrothermal vents (9, 10).

The phylum *Planctomycetota* is still underrepresented in terms of axenic cultures, and only the basic principles of their uncommon cell biology and physiology have been investigated to date (11–13). Taxonomically, the current phylum is subdivided into the four classes *Planctomycetia*, *Phycisphaerae*, “*Ca*. Brocadiia,” and “*Ca*. Uabimicrobiia” (14–17). Research on cell biology focused on differences in the cell division mechanisms (18) and an unusual Gram-negative cell envelope architecture (19). While members of the class *Planctomycetia* reproduce asymmetrically by “budding” (20, 21), members of the other classes divide by binary fission (15, 17, 22–24). Several members of the class *Planctomycetia* perform a lifestyle switch: surface-attached mother cells give rise to flagellated daughter cells (25, 26) that either swim away or join the biofilm.

All known planctomycetes lack most canonical bacterial cell division genes except for the DNA translocase-encoding gene *ftsK* (27, 28). The mechanistic principles of their cell division are still poorly understood (23).

In this study, we combined a cultivation approach targeting the isolation of novel members of the phylum *Planctomycetota* and cultivation-independent 16S rRNA gene amplicon sequencing to elucidate the bacterial diversity in a shallow-sea hydrothermal vent close to the island Panarea (Italy) in the Tyrrhenian Sea. The chosen sampling location is characterized by increased temperatures and elevated concentrations of trace elements and complex sulfur sources (29).

## MATERIALS AND METHODS

### Sample collection and processing

Water samples from the transition zone (38.6401 N 15.1097 E, temperature: 27.6°C, pH 5.62) and the surface (38.6384 N 15.1067 E, temperature: 26.7°C, pH 8.01) of the shallow-sea hydrothermal vent close to Panarea Island were collected on 11th September 2013 in sterile polypropylene bottles and immediately transferred to the expedition laboratory. The samples were first run through a glass fiber filter with a pore size of 2.7 µm, followed by filtration using 0.22 µm polycarbonate filters. The filters were stored at −20°C until DNA extraction. Gelatinous material was scrubbed from a rocky overhang 30 cm above the water surface, collected in a sterile polypropylene bottle, and stored at 4°C until cultivation.

### DNA extraction and whole genome amplification

DNA from water filters was extracted using the PowerBiofilm DNA Isolation Kit (MoBio Laboratories, Dianova) following the manufacturer’s protocol with the following exceptions: (i) the time of incubation at 37°C in buffer B1 was increased to an overnight step, (ii) incubation at 55°C was increased to 30 min, and (iii) incubation at 4°C was increased to 20 min. Bead-beating was performed in a FastPrep-24 instrument (MP Biomedicals) at 5.5 m/s for 30 s. The DNA was eluted in 100 µL BF7 buffer and stored at −20°C until further processing. Genomic DNA extracted from water filters was amplified by multiple displacement amplification (MDA) based on phage Ф29 (phi29) DNA polymerase (30). For this purpose, the illustra GenomiPhi V3 DNA Amplification Kit (GE Healthcare) was used following the recommendations of the manufacturer. For one single amplification reaction (20 µL total volume), 1 ng of genomic DNA was used. To reduce remaining stochastic amplification bias, three independent reactions per filter were pooled. To reduce contamination with external DNA, preparation steps not involving a DNA template were performed in a PCR cabinet (AirClean Systems, StarLab, Hamburg, Germany) previously decontaminated using DNA-away (Molecular BioProducts, Thermo Fisher Scientific, Waltham, USA) and UV light for 1 h. The template DNA was added in another room in a second PCR cabinet of the same brand, and amplification reactions were performed in a PCR cycler (Veriti 96-Well, Applied Biosystems). MDA-amplified gDNA was stored at −20°C until further processing.

### Amplicon preparation

Amplification of variable region 3 (V3) of 16S ribosomal RNA genes was performed using two subsequent PCR amplifications. The first protocol was used to enrich the V3 region of MDA DNA obtained from the filters. In this protocol, the universal forward primer 341f (5′-CCT ACG GGW GGC WGC AG-3′) and reverse primer uni515r (5′-CCG CGG CTG CTG GCA C-3′) (modified from 518r) (31) were used. The second PCR protocol was then performed with extended V3 region primers V3F (5′-AAT GAT ACG GCG ACC ACC GAG ATC TAC ACT CTT TCC CTA CAC GCT CTT CCG ATC TCC TAC GGG WGG CWG CAG-3′) and indexed V3R primers (5′-CAA GCA GAA GAC GGC ATA CGA GAT XXX XXX GTG ACT GGA GTT CAG ACG TGT GCT CTT CCG ATC TCC GCG GCT GCT GGC AC-3′) modified from a previous protocol (32). PCRs for the first protocol of 50 µL contained 25–29 µL microbial DNA-free water (Qiagen, Venlo, Netherlands), 10 µL 5× Q5 Reaction Buffer (final conc. 1×; New England Biolabs), 10 µL 5× Q5 High GC Enhancer (final conc. 1×; New England Biolabs), 1 µL dNTP Mix (final conc. 200 µM; New England Biolabs), 0.5 µL of each primer (341f, uni515r; final conc. 0.1 µM), 0.5 µL Q5 High Fidelity DNA Polymerase (final conc. 0.02 U/µL; New England Biolabs), and 1–5 µL amplified MDA gDNA (~500 ng). The cycling program consisted of an initial denaturation step at 94°C, 5 min, followed by 10 cycles of denaturation at 94°C, 1 min, annealing at 63°C, 1 min, elongation at 72°C, 1 min, and a final elongation step at 72°C, 10 minutes. Three independent pre-amplification reactions were pooled and stored at 4°C until further processing. The next PCR amplification was performed to add sequence indices and adapter sequences for subsequent Illumina sequencing. PCRs of 50 µL contained 13.1 µL PCR-grade H_2_O (Qiagen, Venlo, Netherlands), 10 µL 5× Q5 Reaction Buffer (final conc. 1×; New England Biolabs), 10 µL 5× Q5 High GC Enhancer (final conc. 1×; New England Biolabs), 1 µL dNTP Mix (final conc. 200 µM; New England Biolabs), 0.2 µL of each primer (V3f, V3_34r; final conc. 0.2 µM), 0.5 µL Q5 High Fidelity DNA Polymerase (final conc. 0.02 U/µL; New England Biolabs), and 10 µL PCR product of the first PCR as DNA template. The amplification was performed with a cycling program including an initial denaturation step at 98°C, 5 min, followed by 10 cycles of denaturation at 98°C, 1 min, annealing at 65°C, 1 min, elongation at 72°C, 1 min and a final elongation step at 72°C, 5 minutes. To reduce stochastic amplification bias, three independent amplifications were performed.

### Amplicon gel electrophoresis and extraction

Amplicon PCR products were separated by agarose gel electrophoresis (2% [wt/vol]; Serva, Heidelberg, Germany) in 1× Tris-acetate-EDTA (TAE) buffer (diluted from 50× TAE with deionized water; Applichem, Darmstadt, Germany) at 130 V for 90 min. The gel was stained with SYBR gold nucleic acid gel stain (final conc. 1× in 1× TAE; Thermo Fisher Scientific, Waltham, USA) for 60 min, and the DNA was visualized by UV light through a blue transilluminator to avoid damaging the DNA. Amplicon bands (fragment size of ~300 bp) were cut out using sterile single-use scalpels and extracted using the NucleoSpin Gel and PCR Clean-up kit (Macherey-Nagel, Düren, Germany). The three replicates of each sample were purified using the same column. Reads of V3 amplicons were obtained from Illumina multiplex sequencing (MiSeq Analyzer). Demultiplexed MiSeq sequence data were processed using the Quantitative Insights Into Microbial Ecology (Qiime2) tool with default parameters (https://docs.qiime2.org). Forward and reverse reads were joined, chimera-filtered, and clustered using the vsearch plugin (33). Joined reads were quality-filtered (34) and trimmed to 165 bp (deblur/denoise: min size 2, min reads 10 [35]). The resulting multi-fasta files were analyzed using SILVAngs v. 1.4.9 with SILVA database v. r138.1 (36).

### Culture media and bacterial strain isolation

M1H NAG ASW medium (M1 medium buffered with 4-(2-hydroxyethyl)−1-piperazineethanesulfonic acid [HEPES] and supplemented with *N*-acetylglucosamine [NAG] and artificial seawater [ASW]) was prepared as previously described (11). Liquid medium was mixed with 12 g/L agar and supplemented with 100 mg/L carbenicillin for the initial incubation of sampled material on agar plates (19). The sampled gelatinous material was homogenized, and dilutions of 1:10 and 1:100 were streaked on solidified M1H NAG ASW agar plates that were incubated at 20°C in the dark for 2 months. Single colonies were streaked on fresh solid M1H NAG ASW agar plates supplemented with carbenicillin. Pure cultures were cryopreserved in M1H NAG ASW medium supplemented with 50% (vol/vol) glycerol or 5% (vol/vol) dimethyl sulfoxide (DMSO) and stored at −80°C. For the cultivation of *Planctopirus limnophila, Gemmata obscuriglobus,* and *Algisphaera agarilytica,* M1H NAG ASW medium was used. “*Candidatus* Kuenenia stuttgartiensis” was obtained from an 80% enrichment of a 2 L batch reactor (37). Growth of the novel isolate, strain Pan216^T^, under microoxic conditions was determined after 17 days with a candle-anaerobic jar system on M1H NAG ASW agar. The jar was incubated at 28°C. A cultivation under anaerobic conditions was performed in M1H NAG ASW S medium (M1H NAG ASW medium, pH 7.5, supplemented with 0.1% [wt/vol] sodium resazurin solution, 1 g/L sulfur [powdered] and 0.23 g/L Na_2_S) for up to 1 month at 28°C.

### Identification of the novel isolate by sequencing of the 16S rRNA gene

Strain Pan216^T^ was identified by direct sequencing of the 16S rRNA gene as previously described (38).

### Genome sequencing and nucleotide sequence accession numbers

Sequencing of the genome of strain Pan216^T^ is part of a previously published study (11). The 16S rRNA gene of the novel isolate is available from GenBank under accession number MK559983. The genome sequence can be found under accession number CP036279.

### Phylogenetic inference

The reconstruction of phylogenetic trees based on 16S rRNA gene sequences and multi-locus sequence analysis (MLSA) was performed as previously described (39). The genomes of three members of the class *Phycisphaerae*, *Phycisphaera mikurensis* FYK2301M01^T^ (acc. no. AP012338.1), *Poriferisphaera corsica* KS4^T^ (acc. no. CP036425.1), and *Algisphaera agarilytica* 06SJR6-2^T^ (acc. no. GCA_014207595.1), were used as outgroup for the MLSA-based tree. Average amino acid identity (AAI) and average nucleotide identity (ANI) values were calculated using scripts of the enveomics collection (40). The percentage of conserved proteins (POCP) was calculated as described (41). The *rpoB* nucleotide sequences (encoding the β-subunit of the RNA polymerase) were taken from publicly available genome annotations, and the sequence identities of a ca. 1300 bp partial sequence were determined as described (42).

### Light microscopy

Cells of strain Pan216^T^ were immobilized on a 1% (wt/vol) agarose pad in MatTek Glass Bottom Microwell Dishes (35 mm dish, 14 mm microwell with No. 1.5 cover-glass P35G-1.5-14-C) and were imaged with phase-contrast or differential interference contrast illumination using a Nikon Ti microscope at ×100 magnification with a Nikon N Plan Apochromat λ 100×/1.45 Oil objective and the Nikon DS–Ri2 camera. To determine the cell size of the novel strains, 100 individual cells were measured using the NIS-Elements software V4.3 (Nikon Instruments). For time-lapse microscopy, images were taken either using the CellASIC ONIX Microfluidic Platform (Merck Millipore) or a 1% (wt/vol) agarose pad supplemented with the corresponding medium as previously described (43). In the microfluidic platform, cells are elastically trapped in a CellASIC ONIX Microfluidic Plate B04 (Merck Millipore) and supplied with a constant flow of liquid medium. Bacterial growth was observed for 4 days. Micrographs were subsequently aligned and analyzed using the NIS-Elements imaging software V4.3 (Nikon Instruments).

### Electron microscopy techniques

Field emission scanning electron microscopy (SEM) was performed based on a previously published protocol (19). Cells of *P. limnophila*, strain Pan216^T^, *G. obscuriglobus,* and *A. agarilytica* were fixed in 1% (vol/vol) formaldehyde in HEPES buffer (3 mM HEPES, 0.3 mM CaCl_2_, 0.3 mM MgCl_2_, 2.7 mM sucrose, pH 6.9) for 1 h on ice and were washed once with 3 mM HEPES buffer. Cover slips with a diameter of 12 mm were coated with a poly-L-lysine solution (Sigma-Aldrich) for 10 min, washed in distilled water, and air-dried. 50 µL of the fixed bacteria solution was placed on a cover slip and allowed to settle for 10 min. Cover slips were then fixed in 1% (vol/vol) glutaraldehyde in TE buffer (20 mM TRIS, 1 mM EDTA, pH 6.9) for 5 min at room temperature and subsequently washed twice with TE–buffer before dehydrating in a graded series of acetone (10%, 30%, 50%, 70%, 90%, and 100%) on ice for 10 min at each concentration. Samples from the 100% acetone step were brought to room temperature before placing them in fresh 100% acetone. Samples were then subjected to critical point drying with liquid CO_2_ (CPD 300, Leica). Dried samples were covered with a gold/palladium (80/20) film by sputter coating (SCD 500, Bal-Tec) before examination in a field emission scanning electron microscope (Zeiss Merlin) using the Everhart Thornley HESE2 detector and the inlens SE detector in a 25:75 ratio at an acceleration voltage of 5 kV. TEM micrographs were taken as previously described (44) after negative staining with 0.1%–2% aqueous uranyl acetate and employing an EM 910 electron microscope (Carl Zeiss) at an acceleration voltage of 80 kV. For cryoSEM sample preparation, cells were placed between two cryostubs forming a sandwich and plunge-frozen in liquid nitrogen slush. Samples were then placed into a cryotransfer system (Gatan Alto 2500; Oxford, United Kingdom). The top cryostub was fractured by a razor. The water layer was sublimated for 10 min at −80°C, sputter-coated with a thin (~2 nm) layer of Au-Pd (60/40 ratio) for 45 s using a Cressington 208 HR sputter coater fitted with an MTM-20 thickness controller (Cressington Scientific Instruments Ltd., United Kingdom). The analysis was performed using a Jeol 6330 cryoscanning electron microscope (Jeol, Tokyo, Japan). Thin sections were prepared by high-pressure freezing and freeze substitution as previously described (25, 45). Sections were subsequently analyzed employing a JEOL 1200EX—80kV TEM microscope.

### Physiological analyses

Temperature, pH, NaCl, and ASW concentration optima for growth were determined by optical density measurements of growing cultures at 600 nm (OD_600_). The strain was inoculated 1:10 from an early stationary phase culture in glass test tubes with M1H NAG ASW medium and incubated under constant agitation in temperature-controlled shakers. Measurements were performed in biological triplicates, and each tube served as its blank prior to inoculation. To determine the pH optimum for growth, M1H NAG ASW medium was buffered to pH values of 5.0, 6.0, 6.5, 7.0, 7.5, 8.0, 8.5, 9.0, and 10.0 using 10 mM of either 2-(*N*-morpholino)ethanesulfonic acid (MES), HEPES, 3-[4-(2-hydroxyethyl)piperazin-1-yl]propane-1-sulfonic acid (HEPPS), or *N*-cyclohexyl-2-amino-ethanesulfonic acid (CHES) buffers, corresponding to their individual buffer range. To determine the salt tolerance, NaCl concentrations ranging from 0% to 10% (wt/vol) or ASW concentrations in the range of 0%–225% in M1H NAG ASW medium were tested. Catalase activity was determined by bubble formation after the addition of a fresh 3% (vol/vol) H_2_O_2_ solution. Cytochrome oxidase activity was determined using Bactident Oxidase test stripes (Merck Millipore) following the manufacturer’s instructions. Substrate utilization experiments were performed as described (46). Cells were incubated for 3–4 weeks at 28°C in the presence of 114 different nutrients using a defined medium containing 2.38 g/L HEPES, 20 mL/L basal salt solution, 250 mL/L ASW, and 5 mL vitamin solution. For fatty acid analysis, cells were grown in liquid M1H NAG ASW medium (pH 8.0) for 7 days at 28°C. Fatty acids were subsequently extracted and analyzed according to the standard protocols of the Microbial Identification System, MIDI Inc.; 6.1 (47). Individual fatty acids were identified using the TSBA40 library (MIDI). APIZYM (bioMérieux) was used based on the manufacturer’s instructions and was examined after 24 h of incubation.

### Circular genome plot

The different core genomes depicted in the circular plot are determined from the results of the Proteinortho analysis (48). All genes predicted to belong to the core genome of all planctomycetes, to the core genome of all budding or the core genome of the non-budding planctomycetes had to be present in strain Pan216^T^ and in at least 91% of the genomes belonging to the respective group (all, budding, binary fission). The COG categories were determined with eggnog-mapper v1.0.3 (49), and the genetic islands were determined with IslandViewer 4 (50). The genome plot was assembled using BRIG (51).

### Identification of S-layer protein homologs

The analysis was based on genes coding for known S-layer proteins of 11 bacteria (52) and archaea, including the S-layer protein of “*Ca*. Kuenenia stuttgartiensis” (locus tag kustd1514). Proteins encoded by strain Pan216^T^ were compared against the known S-layer proteins using BLASTp. Significant results were used for a reciprocal BLAST against the NCBI database and the query organisms. Detected proteins were then analyzed for pI, export signal, subcellular localization, and hydrophobicity. Homologous proteins required an identity >30%, an e-value lower than 1E-6, and a conserved domain architecture. pI values were provided by ExPASy (53). Export signals were predicted using the SecretomeP 2.0 Server. Protein localization was analyzed using PSORTb 3.0.2. Protein domains were analyzed using InterPro (54). Finally, we examined the candidate proteins for the presence of domains required for S-layer attachment to the secondary cell wall polymers.

## RESULTS

### Cultivation-independent amplicon sequencing analysis

**Fig 1:**
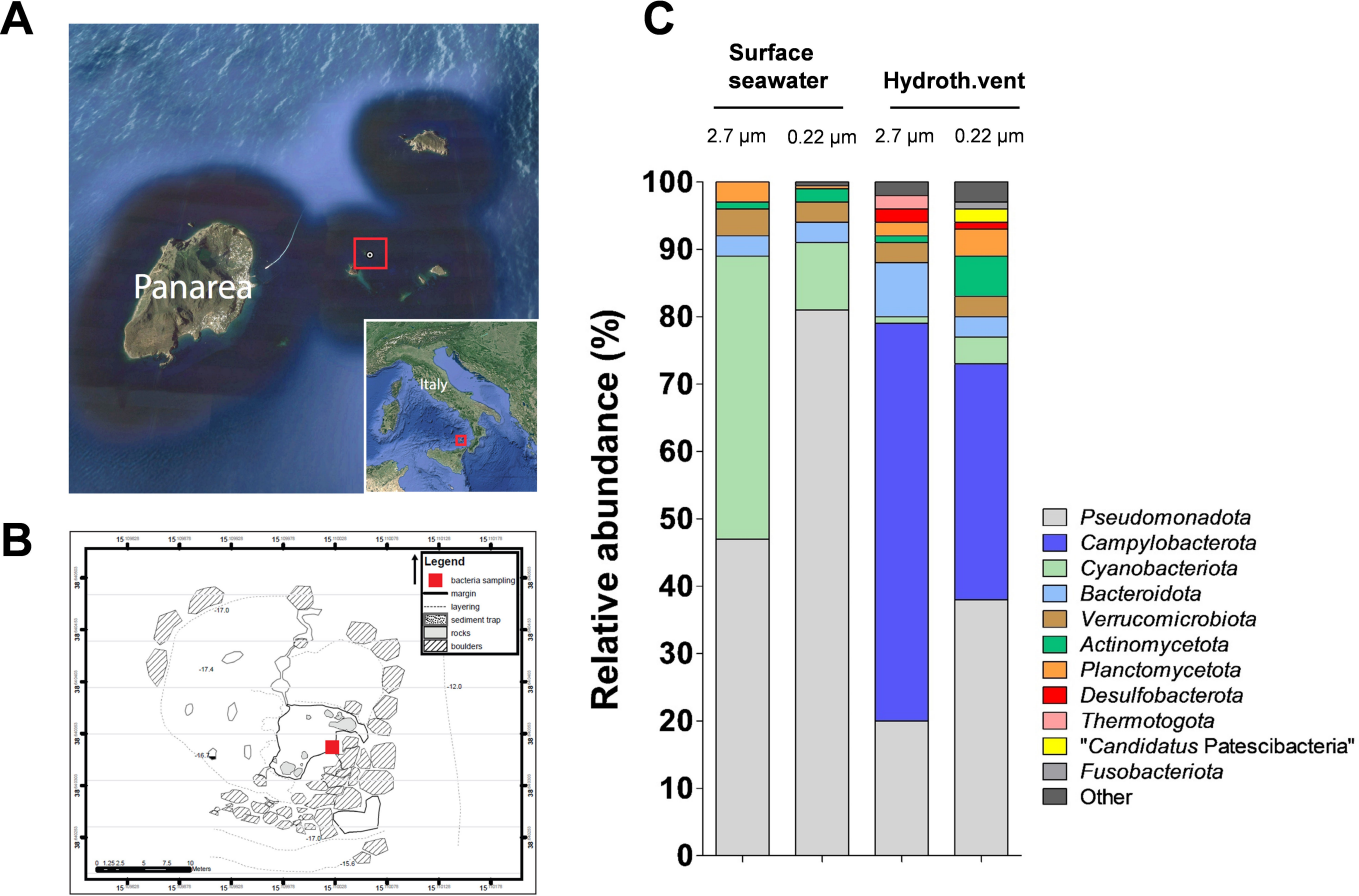
Sampling site and bacterial community composition in the hydrothermal vent system close to Panarea using 16S rRNA (**V3**) amplicon sequencing. (**A**) Sampling site in Panarea (Italy). The map was created with material from OpenMapTiles (obtained via https://satellites.pro/). (**B**) Diving map of the sampling site. The map is a hand-drawn illustration. (**C**). Bacterial community composition in surface seawater and in the transition zone of a hydrothermal vent was analyzed using fractions obtained by filtration with glass fiber (2.7 µm) and polycarbonate (0.22 µm) filters (*n* = 2).

To investigate the on-site composition of bacterial communities, we took samples in the shallow water hydrothermal vent system close to the island Panarea in the Tyrrhenian Sea (Fig. 1A and B). Water samples were taken from the transition zone of the vent at the border line between anaerobic nutrient-rich areas and the oxic surrounding seawater. Surface seawater served as a reference. Samples from both locations were filtered through a 2.7 µm glass fiber filter (for collection of the attached fraction), followed by a filtration through a 0.22 µm polycarbonate filter (for collection of the free-living fraction). 16S rRNA gene amplicon sequencing results of the collected attached and free-living fractions from the two sampling spots (surface water and hydrothermal vent transition zone) were analyzed separately (Fig. 1C).

In the surface water samples, members of the phylum *Pseudomonadota* (formerly *Proteobacteria*) showed the highest relative abundance, which was higher in the free-living fraction (81%) than in the attached fraction (47%). *Cyanobacteria* were the second highest abundant phylum in both fractions of the seawater sample, which is not unexpected when considering that photosynthetic strains inhabit the light-exposed surface waters. In line with this observation, members of this phylum were found in lower abundance in the transition zone (1%–4%). The transition zone close to the hydrothermal vent was dominated by *Pseudomonadota* (and *Campylobacterota*, now regarded as a separate phylum [55]) (Fig. 1C). In total, amplicon sequences could be assigned to seven different phyla in the seawater samples, whereas a greater diversity of 20 different phyla occurred in the transition zone. In the latter, even rare phyla such as *Ignavibacteriota*, “*Candidatus* Parcubacteria,” and “*Candidatus* Gracilibacteria” could be detected in a relative abundance of up to 1%. The total number of unique operational taxonomic units (OTUs) was nearly twice as high in the transition zone compared to the seawater (Table S1), supporting the notion of higher diversity there. In addition, a weighted UniFrac analysis showed a considerable difference between the two sampling locations, pointing toward different bacterial communities. This finding becomes even more evident by comparing the most abundant OTUs at the family and genus levels (Table S1). In the transition zone, *Sulfurimonas*, *Sulfurovum* (both from the phylum *Campylobacterota*), and other genera known to grow anaerobically had the highest relative abundances. By contrast, the seawater control contained mostly members of the provisional order “*Candidatus* Pelagibacterales*”* (α-proteobacteria, formerly SAR11 clade) or different cyanobacteria.

Since we were particularly interested in members belonging to the phylum *Planctomycetota*, we investigated amplicon sequences of members of this phylum in greater detail. *Planctomycetota* showed a relative abundance of up to 3.9% in the transition zone (free-living fraction) and of 2.9% in the seawater control (attached fraction). Within the attached- and free-living fraction of the hydrothermal vent sample, one specific OTU (43% and 83%, respectively) was highly enriched. BLAST analysis of the partial 16S rRNA gene sequence showed a 97% sequence similarity to *Thermostilla marina* (family *Thermoguttaceae*). *T. marina* is a thermophilic and facultatively anaerobic species. The type strain SVX8^T^ was isolated from the shallow submarine hydrothermal vent close to Vulcano Island (56).

The planctomycetal diversity in the free-living fraction of the seawater sample and the attached fraction of the hydrothermal vent sample was higher than in the other two samples. Unfortunately, the current version of the SILVA database has not yet included most of the ca. 60 new genera and ca. 120 new species described in the last 10 years. Instead, it is restricted to 2–3 genera per family. Hence, the automated genus assignment performed by the SILVAngs pipeline does not achieve a sufficient resolution to reflect the currently explored planctomycetal diversity at the genus and species levels. To improve the resolution, all unique sequences with “*Planctomycetota”* as assigned phylum were extracted from all samples and used for the construction of a single phylogenetic tree, together with all currently described members of the phylum (Fig. S1). The tentative assignment to described genera was performed manually based on the clustering pattern and branch lengths of the tree (Table S2). In the tree, most amplicon sequences clustered in the orders *Pirellulales* and *Planctomycetales*. Only a single sequence was found that belonged to a member of the order *Gemmatales*, while no member of the order *Isosphaerales* could be detected in either of the samples. Except for a single exception each, members of the class *Phycispherae* and “*Saltatorellus* clade” (OM190 lineage) were exclusively found in the transition zone samples.

In the entire data set for the phylum *Planctomycetota*, the genera with the highest number of members were *Mariniblastus* (19 sequences), *Stieleria* (15 sequences), and *Botrimarina* (12 sequences) for the order *Pirellulales,* and *Fuerstiella* (10 sequences) and *Rubinisphaera* (6 sequences) for the order *Planctomycetales* (Table S2). The data set suggests several novel planctomycetal genera in the samples, including members of the shapeshifting “*Saltatorellus* clade” (Fig. S1) (57). Thus, the hydrothermal vent system of the Eolian Archipelago close to Panarea represents a promising source for the isolation of novel members of the phylum *Planctomycetota*, which is reinforced by the isolation of several novel strains from this location in previous studies (56, 58–60).

### The novel planctomycetal isolate strain Pan216^T^ belongs to a novel family

The results of the cultivation-independent analyses indicated a moderate abundance of *Planctomycetota* (up to 4%, rank 4 of 23 in the free-living fraction of the transition zone) with a largely uncultivated diversity in this location. Thus, we followed an optimized procedure for the isolation of novel *Planctomycetota* (11). Cultivation of samples from gelatinous material found at the sampling site led to the isolation of the novel strain Pan216^T^, for which we analyzed phylogeny as well as phenotypic and genomic features. The phylogenetic inference was based on five different phylogenetic criteria (16S rRNA gene sequence identity, ANI, AAI, POCP, and partial *rpoB* sequence identity) and included all characterized planctomycetal species available as of June 2024.

**Fig 2:**
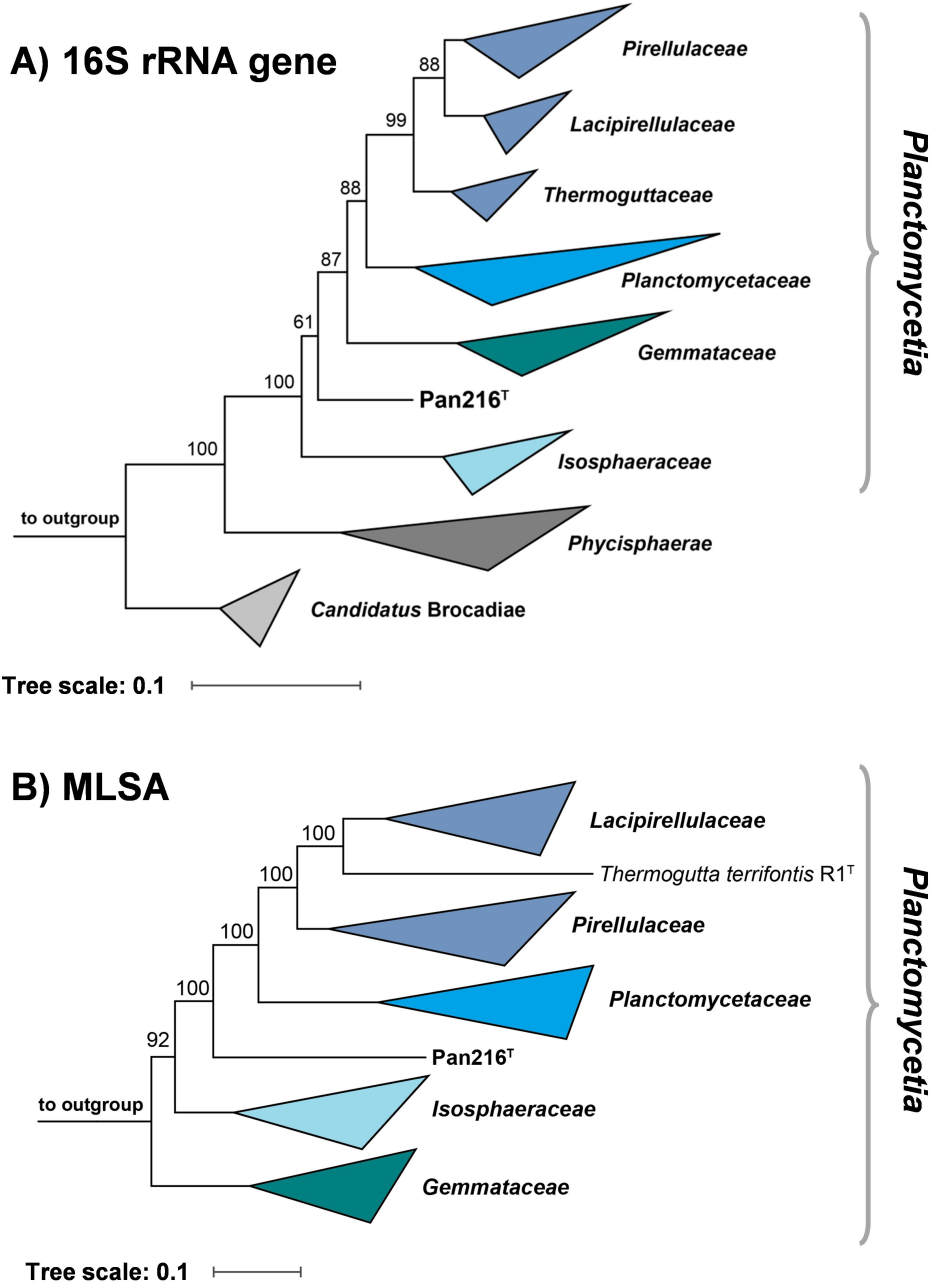
Maximum likelihood phylogenetic analysis. Phylogenetic trees showing the position of strain Pan216^T^. The 16S rRNA gene sequence- (**A**) and multilocus sequence analysis (MLSA)-based phylogeny (**B**) was computed as described in the Materials and Methods section. Bootstrap values after 1,000 re-samplings (16S rRNA gene)/500 re-samplings (MLSA) are given at the nodes (in %). Three 16S rRNA genes of bacterial strains outside of the phylum *Planctomycetes*, but part of the PVC superphylum, were used as outgroup in the 16S rRNA gene sequence-based tree (NCBI acc. no. AJ229235, CP010904.1 and NR_027571). The genomes of three members of the class *Phycisphaerae*, *Phycisphaera mikurensis* FYK2301M01^T^ (acc. no. AP012338.1), *Poriferisphaera corsica* KS4^T^ (acc. no. CP036425.1), and *Algisphaera agarilytica* 06SJR6-2^T^ (acc. no. GCA_014207595.1), were used as outgroup in the MLSA-based tree.

In the phylogenetic tree based on 16S rRNA gene sequences, strain Pan216^T^ clustered on a separate branch between the families *Gemmataceae* and *Isosphaeraceae* (Fig. 2A). In the MLSA-based tree, strain Pan216^T^ formed a separate branch between the families *Isosphaeraceae* and *Planctomycetaceae* (Fig. 2B). Based on the phylogenetic trees, no closely related genus or family for strain Pan216^T^ could be detected. The novel isolate showed the highest 16S rRNA gene sequence similarity (85.4% identity) to *Thermostilla marina* (family *Thermoguttaceaea*, order *Pirellulales*), suggesting this species as the closest relative. This value falls below the family threshold of 86.5% (61), supporting the separation of strain Pan216^T^ from known families. Unfortunately, the genome of *T. marina* has not been sequenced, which hinders comparison of the strains using whole genome-based markers; we did not pursue a whole-genome comparison with the next most closely related genome-sequenced strain (*Thermogutta terrifontis*), as it is more distantly related (84.6% identity to Pan216^T^) and ranked only 7th of the most similar strains based on their 16S rRNA gene sequences.

The three closest related families *Isosphaeraceae*, *Gemmataceae,* and *Planctomycetaceae* belong to different orders in the class *Planctomycetia,* and the placement of strain Pan216^T^ between the families suggests it is a member of this class. For the other analyzed phylogenetic criteria, such as AAI, we observed the highest similarity of 42.8% when comparing strain Pan216^T^ with the type strain of *Gimesia maris*. This value falls significantly below the threshold of 60% for delineation of genera (62), which would indicate a relationship at least on the genus level. A partial sequence of the gene *rpoB* encoding the β-subunit of RNA polymerase is used as a phylogenetic marker for the family *Planctomycetaceae* (42), and a genus threshold range of 75.5%–78% was proposed for the reorganized order *Pirellulales* (63, 64). For strain Pan216^T^, we obtained the highest similarity of the partial *rpoB* sequence of 74.1% with *Aquisphaera giovannonii* (family *Isosphaeraceae*) (65). The observed similarity is below the above-mentioned genus threshold, which is in line with our conclusions based on AAI and 16S rRNA gene sequence similarity (as well as ANI and POCP values that suggested additional strains as the current closest neighbors of the novel isolate). Taken together, all analyzed markers support the separation of strain Pan216^T^ at least from the known genera. The maximal 16S rRNA gene similarity below the family threshold even separates the strain from known families. Phylogenetic inference thus points toward the classification of strain Pan216^T^ as representative of a novel species in a novel genus of a thus far uncharacterized family.

### Morphological and physiological characterization of strain Pan216^T^

**Fig 3:**
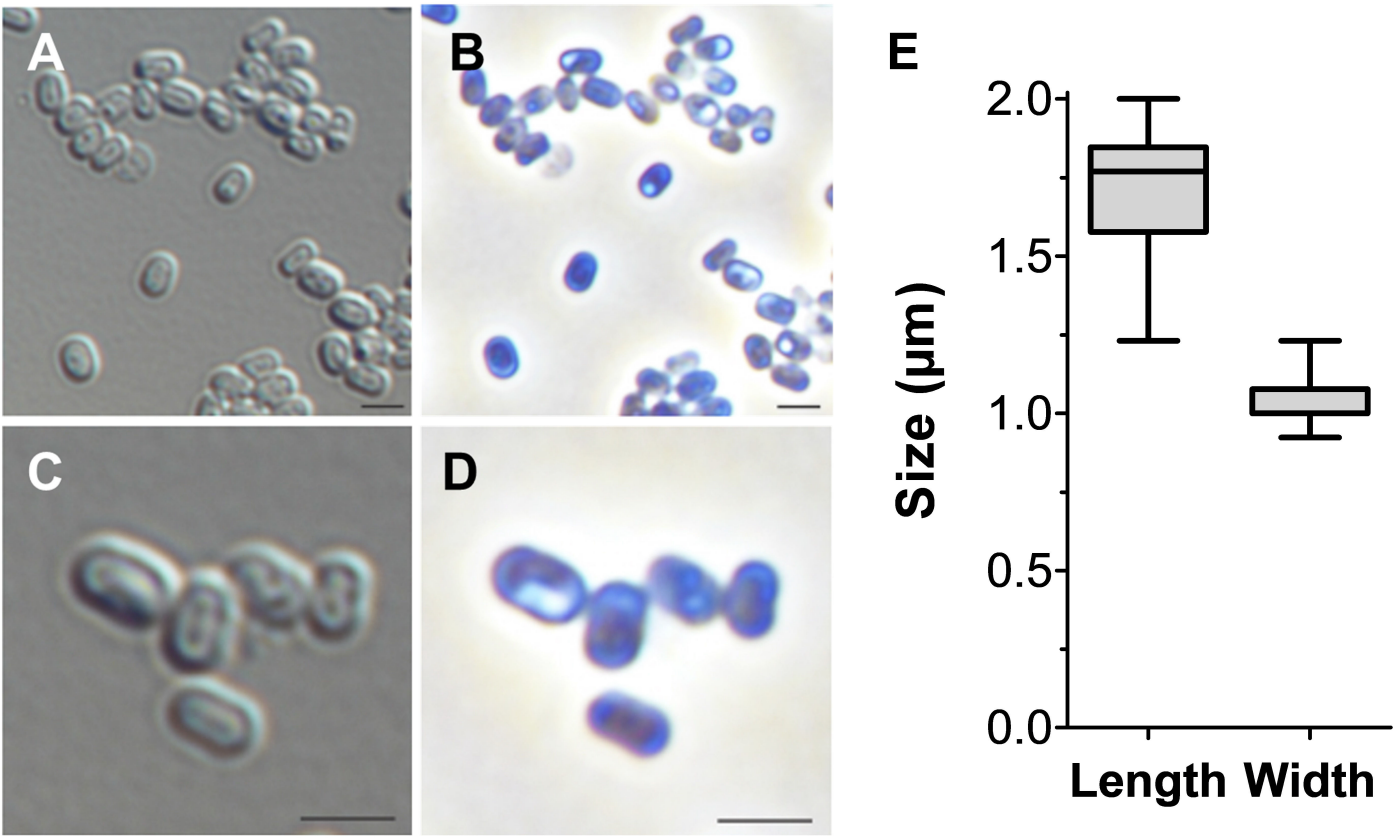
Cell morphology and cell size of Pan216^T^. The morphology and average cell size of strain Pan216^T^ were determined by light microscopy with differential interference contrast (DIC, **A and C**) and phase-contrast (Phaco, **B and D**) illumination. Cells of strain Pan216^T^ are of oblong, cylindrical morphology and do not grow in clusters under aerobic conditions. Cell size was determined by measuring 100 individual cells (**E**). Scale bar 2 µm.

**Fig 4:**
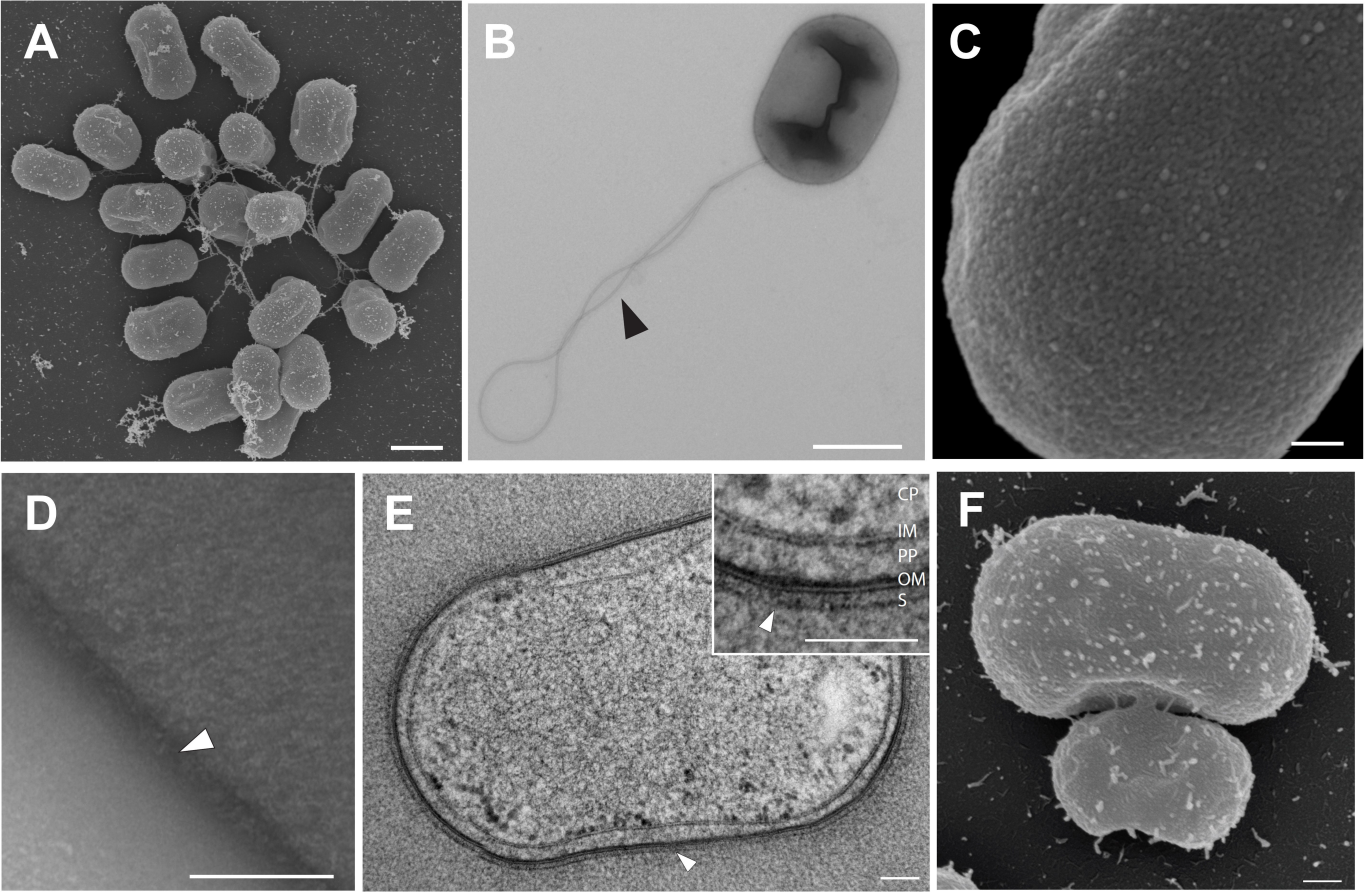
Morphological characteristics of strain Pan216^T^. (**A**) SEM overview of Pan216^T^ showing an oblong-shaped cell. (**B**) TEM image of a flagellated (black arrowhead) Pan216^T^ cell. (**C**) SEM image of the surface of Pan216^T^ depicting an unusual cell surface. (**D**) TEM image of the outer surface of Pan216^T^ revealing an additional structure (white arrowhead) surrounding the cell. (**E**) Thin section of a high-pressure frozen and freeze-substituted Pan216^T^ cell. The inset gives a detailed view of the membrane organization, visualizing the cytoplasm (CP), the inner membrane (IM), the periplasm (PP), the outer membrane (OM), and a putative surface layer (S, white arrowhead). (**F**) SEM micrograph depicting the novel cell division by lateral budding of Pan216^T^. Scale bars: A, B: 1 µm; C, D, and E: 0.1 µm; F: 0.2 µm.

Cells of strain Pan216^T^ have a cylindrical (pill/capsule-like) shape (length 1.7 ± 0.2 µm, width 1.1 ± 0.2 µm) (Fig. 3A, C, and 4) and form white colonies on solid medium. Cells are flagellated, motile (Fig. 4B), do not form a visible stalk, and occur as single cells or form loose aggregates. The presence of inner and outer membranes led to the classification of the cell architecture as Gram-negative (Fig. 4E). SEM and TEM micrographs suggest that the cell surface is not symmetrically structured but harbors an additional outer layer (Fig. 4C and D). During TEM analysis of high-pressure frozen, freeze-substituted, and thin-sectioned cells, a surface layer was detected, surrounding the outer membrane of the cell (Fig. 4E). In comparison to other planctomycetes, no invaginations of the cytoplasmic membrane could be observed for strain Pan216^T^.

During lab-scale cultivation experiments, strain Pan216^T^ was able to grow under aerobic, microaerobic, or anaerobic conditions. Growth was observed over a temperature range of 16°C–34°C with optimal growth at 32°C. The strain grew at a pH range of 6.0–10.0 (optimum 8.0) and an ASW concentration from 10% to 100% with an optimum of 50%. Cells require a minimum concentration of 3.5% (wt/vol) NaCl and showed optimal growth at 7% (wt/vol) NaCl (Fig. S2). A maximal growth rate of 0.035 h^−1^ was observed, corresponding to a generation time of 20 hours.

Cells tested positive for catalase and oxidase activities and formed biomass using a variety of different substrates (Table S3). Good growth was observed with adonitol, fermented rumen extract, gluconate, lactate, maltose, mannitol, melizitose, *N*-acetyl galactosamine, *N*-acetyl glucosamine, ornithine, protocatechuate, raffinose, shikimate, sucrose, trehalose, xylose, and yeast extract. The fatty acid profile was analyzed after growth in M1H NAG ASW medium (pH 8, 7 days cultivation at 28°C), which revealed 14:0, 16:0, 18:1 ω7c as well as 16:1 ω7c, and/or 15/iso-2-OH (which cannot be discriminated using gas chromatography) as the major fatty acids (Table S4). Enzymatic activity determination using the APIZYM system was tested, but replicate assays with strain Pan216^T^ did not generate reproducible or reliable results. Hence, only leucine arylamidase activity could be confirmed.

Comparison of phenotypic and genomic features of strain Pan216^T^ with species from related families (*G. obscuriglobus*, *Isosphaera pallida*, *T. marina*) revealed major differences for many of the investigated features (Table 1). These results provide additional support for classifying strain Pan216^T^ as a member of a novel family.

**TABLE 1:** Comparison of phenotypic and genotypic features of Pan216^T^ with species from closely related families^*a*^^,*b*^

Characteristic	Pan216^T^	*Gemmata obscuriglobus* DSM 5831^T^	*Isosphaera pallida* IS1B^T^	*Thermostilla marina* SVX8^T^
Phenotypic features				
Size (length × width) (µm)	1.7 ± 0.2 × 1.1 ± 0.2	1.4–3.0	2.5–3.0 (diameter)	0.5–1.2
Shape	Cylindrical	Spherical to pear-shaped	Spherical	Ovoid to drop-shaped
Aggregates	Yes	Yes	Yes	Yes
Colony color	White	Rose	Salmon-colored	n.d.
Temperature range (optimum) (°C)	16–34 (32)	16–35	34–55 (41)	32–68 (55)
pH range (optimum)	6.0–10.0 (8.0)	n.d.	n.d. (7.8–8.8)	5.0–9.0 (7.0–8.0)
NaCl range (optimum) (% wt/vol)	4–10 (7)	n.d.	n.d.	0.8–4.5 (2.5–3.5)
ASW range (optimum) (%)	10–100 (50)	n.d.	n.d.	n.d.
Relation to oxygen	Facultatively anaerobic	Aerobic	Obligate aerobic	Anaerobic/microaerobic
Division	Lateral budding	Budding	Budding	Budding
Dimorphic life cycle	n.o.	Yes	n.d.	yes
Flagella	Yes	Yes	Yes	Yes
Crateriform structures	n.o.	n.d.	Ubiquitous	n.d.
Fimbriae	Matrix or fiber	Yes	Yes	Yes
Capsule	S-layer	n.d.	No	n.d.
Stalk	n.o.	No	n.d.	n.d.
Holdfast structure	n.o.	No	no	n.d.
Genomic features				
Genome size (bp)	7,613,473	8,999,201	5,529,304	n.d.
Plasmids	no	no	1	n.d.
DNA G + C content (%)	62.0	67.4	62.5	58.5
Completeness (%)	98.28	94.83	98.28	n.d.
Contamination (%)	0	6.03	0	n.d.
Protein-coding genes	5,766	7,465	3,761	n.d.
Hypothetical proteins	2,292	3,608	1,821	n.d.
Protein-coding genes/Mb	757	830	680	n.d.
Coding density (%)	84.3	84.2	84.7	n.d.
Giant genes (>15 kb)	2	1	0	n.d.
tRNA genes	49	98	51	n.d.
16S rRNA genes	1	5	3	n.d.

^*a*^ n.d. not determined.

^*b*^ n.o. not observed.

### Uncommon cell division of the novel isolate by lateral budding

**Fig 5:**
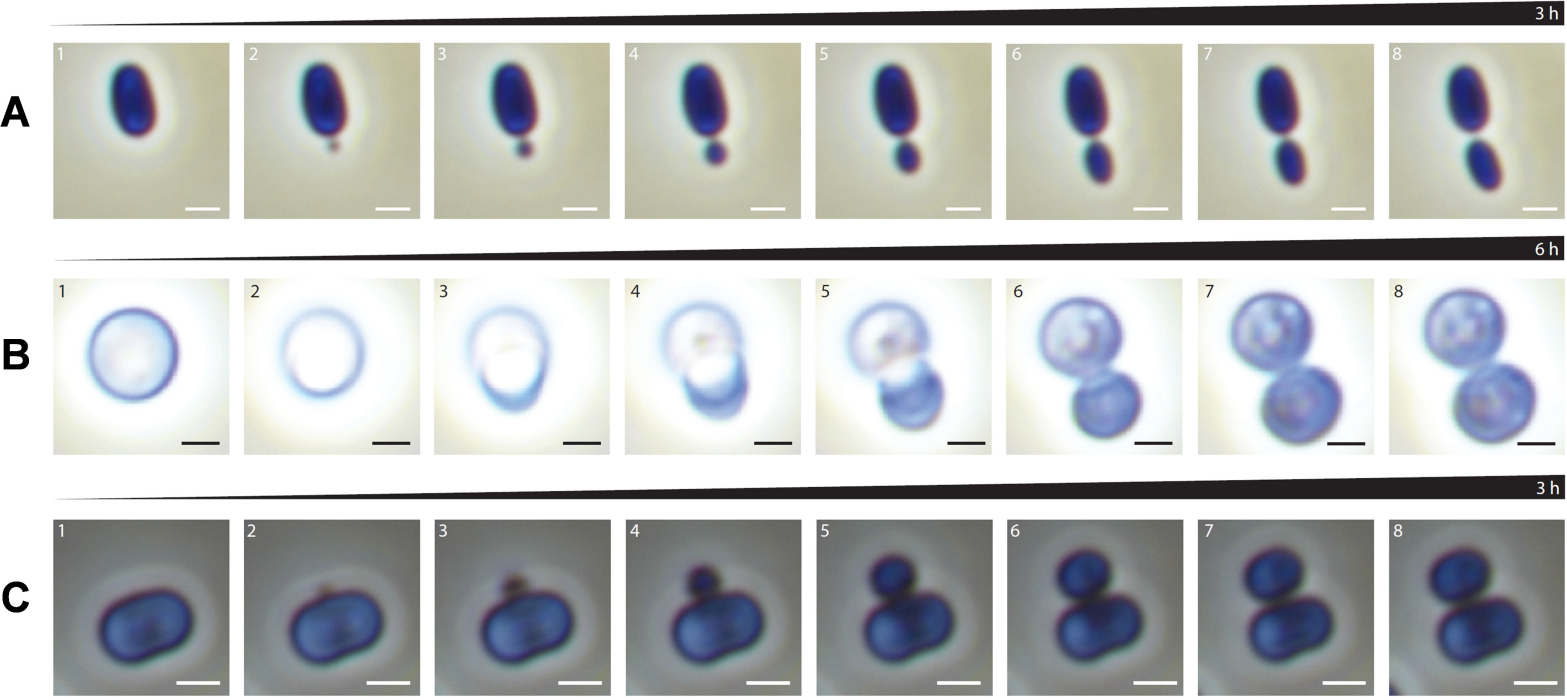
Lateral budding is a novel mechanism of cell division in *Planctomycetota*. (**A**) Time series over 3 h (eight representative images) of the polar budding of *Planctopirus limnophila*. (**B**) Time series over 6 h (eight representative images) of budding of *Gemmata obscuriglobus*. (**C**) Time series over 3 h (eight representative images) of lateral budding of Pan216^T^ shows the formation of the daughter cell at the midcell of the elongated mother cell. Scale bar A, B: 1 µm. (For details, see Movies S1 to S3).

The phylum *Planctomycetota* includes strains performing two different types of cell division—binary fission or budding. While members of the class *Planctomycetia* divide asymmetrically by (polar) budding (Fig. 5A and B; Fig. S3A and B), the spherical species of “*Ca*. Brocadiia” and *Phycisphaerae* perform binary fission (Fig. S3C and D). We reassessed different modes of cell division of different planctomycetal strains using time-lapse microscopy. Asymmetric division of cells of the model strain *P. limnophila* is initiated at the pole (Fig. 5). The daughter cell has a roundish shape at the initial stage of division but reaches the same shape as the mother cell after budding is complete (the daughter cell can be slightly smaller than the mother cell). This process takes approximately 3 h until release from the mother cell (Fig. 5; Movie S1). For spherical cells of *G. obscuriglobus,* the cell division takes about 6 h until detachment (Fig. 4; Movie S2). Daughter cells increase in size and stay attached to the mother cell. During this time, internal changes in the mother and daughter cells could be observed, showing a transfer to the bud (Fig. 5; Movies S1 and S2). In contrast to the budding mode of the current members of the class *Planctomycetia*, strain Pan216^T^ divides by lateral budding (Fig. 4F; Fig. S3E). Time-lapse microscopy revealed that the budding process is initiated exclusively on the elongated side of the cell and takes around 3 h to form the daughter cell (Fig. 5C; Movie S3). During bud formation, the daughter cell is connected to the mother cell via a tubular structure (Fig. 4F). During the early phase of cell division, the cylindrical shape of the mother cells changes to a pill-like shape. Internal changes during reproduction were observed in the mother cell. Components of unknown nature accumulate at the mid-cell and are transferred to the daughter cell (Fig. 5; Movie S3). This transfer process can take up to 24 h after the formation of the daughter cell under the given conditions.

### Genome-based analyses of genes involved in cell division and S-layer formation

**Fig 6:**
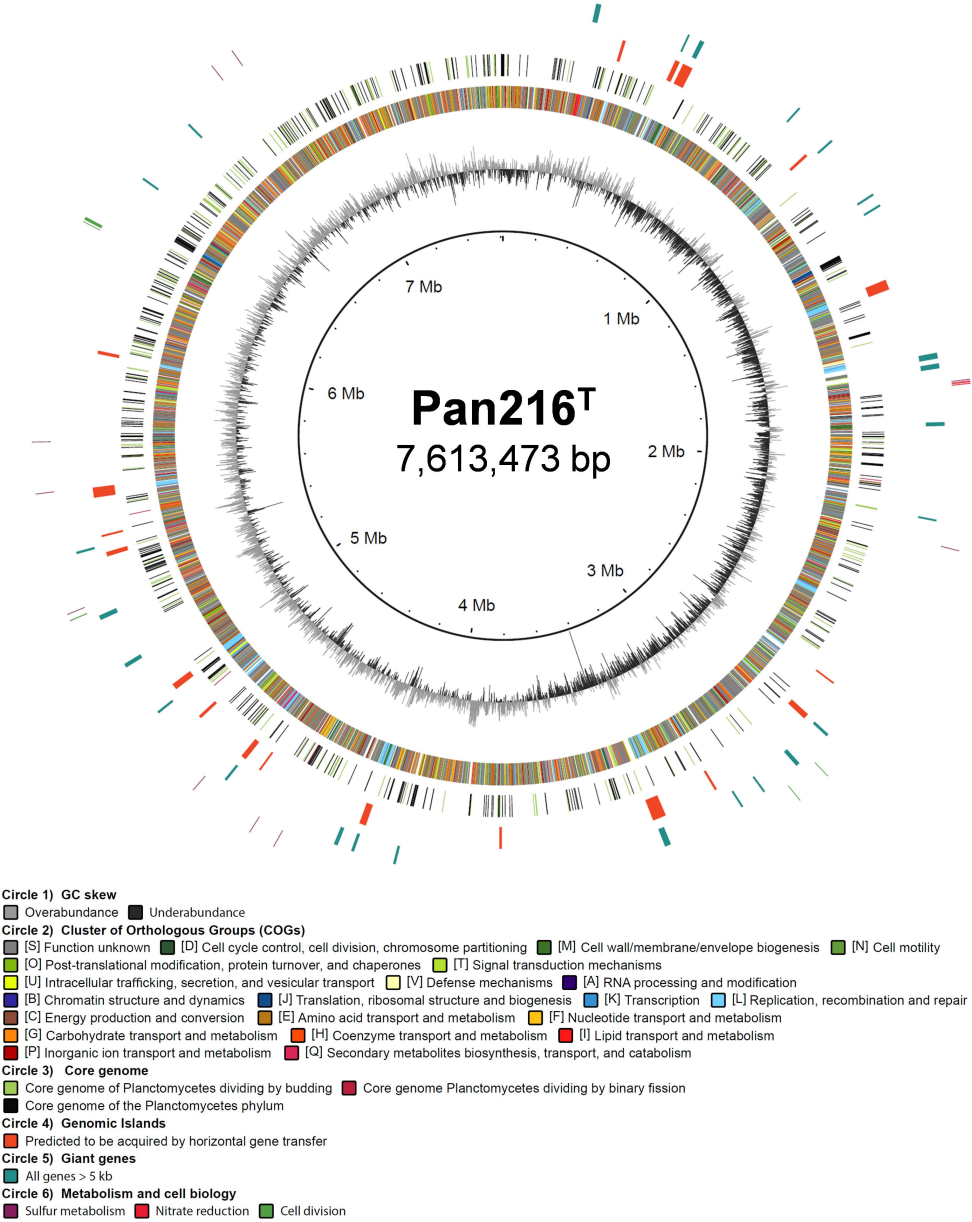
Circular genome plot of strain Pan216^T^. The genome of strain Pan216^T^ is visualized by six individual circles. The innermost circle depicts the G + C skew in light and dark gray. The second circle shows the color-coded COG (Cluster of Orthologous Groups) categories of the encoded proteins. Circle three illustrates the core genome of strain Pan216^T^ determined by the method of reciprocal best alignment. The given color code illustrates whether a gene belongs to the general Planctomycetes core genome of all analyzed Planctomycetes (green), to the core genome of Planctomycetes dividing by budding (black), or by binary fission (blue). The fourth circle holds those genes predicted to be in genomic islands. Circle five depicts “giant genes”—a term describing all genes longer than 5 kB. The outermost circle contains genes partaking in cell division, sulfur metabolism, and assimilatory nitrate reduction.

Strain Pan216^T^ has a genome size of 7,613,473 bp with a DNA G + C content of 62.0%. The annotation with Prokka revealed the presence of 5,766 putative protein-coding genes, of which 2,292 (40%) are annotated as hypothetical or uncharacterized proteins. Genome features are summarized in Fig. 6 and Table 1. To determine proteins putatively involved in the unusual mode of cell division, a core genome was generated based on the comparison of strain Pan216^T^ with non-budding, budding, or all sequenced planctomycetes (Table S5). We found 5,159 and 449 orthologous groups, respectively. However, inspection of the core set of genes coding for enzymes involved in peptidoglycan synthesis and cell division (*mur* and *fts* genes) could not give us any clear hints on differences in the cell division mode. The following genes involved in cell division and peptidoglycan synthesis were found in the genome of strain Pan216^T^: *murA* (UDP-*N*-acetylglucosamine 1-carboxyvinyltransferase), *murB1/murB2* (UDP-*N*-acetylenolpyruvoylglucosamine reductase), *murC* (UDP-*N*-acetylmuramate--L-alanine ligase)*, murD* (UDP-N-acetylmuramoylalanine--D-glutamate ligase), *murE* (UDP-*N*-acetylmuramoyl-L-alanyl-D-glutamate-−2,6-diaminopimelate ligase), *murF* (UDP-*N*-acetylmuramoyl-tripeptide--D-alanyl-D-alanine ligase), *marZ* (transcriptional regulator), and *ftsK* (DNA translocase) (Table S6).

An S-layer on the surface of Pan216^T^ cells was observed using TEM (Fig. 4E). We also analyzed the genome of the isolate for the presence of genes coding for putative S-layer proteins. The search for homologous proteins was based on known S-layer proteins of 11 bacteria and 5 archaea (Table S7) (52), including the S-layer protein of “*Ca*. Kuenenia stuttgartiensis” encoded by the gene *kustd1514* (66). The analysis yielded a list of candidates (Pan216_39030, Pan216_33330, Pan216_25710, and Pan216_13150). However, elucidation of the exact nature of the S-layer in strain Pan216^T^ will require experimental evidence from future studies.

## DISCUSSION

### Cultivation-independent analyses of bacterial communities

In this study, the composition of bacterial communities in the hydrothermal vent system close to Panarea Island was analyzed on different phylogenetic levels. Samples were taken from the transition zone at the borderline between anaerobic nutrient-rich areas and the oxygenized surrounding seawater. The transition zone represents a unique oxygen-limited environment that is dominated by *Pseudomonadota/Campylobacterota* (accounting for ca. 79% of the attached-living and ca. 73% of the free-living fraction), including the genera *Sulfurimonas* and *Sulfurovum* and other uncharacterized genera. These observations are in line with previous analyses of this geographical location, which describe a narrow range of redox conditions with *Sulfurimonas* and *Sulfurovum* as the most dominant genera (67).

A high content of nutrients, including different sources of carbon, nitrogen, and sulfur, in combination with temperature gradients can explain the observed phylogenetic diversity in this marine habitat (Table S1A through D). Such conditions also appear favorable for planctomycetes, which are found associated with diverse macroscopic and microscopic abiotic or biotic surfaces (7, 68). The tendency to form aggregates can imply lower abundance observed in free-living fractions in aquatic habitats. Although the phylum *Planctomycetota* can account for more than 80% relative abundance in bacterial communities, typical ranges of 3%–10% are observed (6). In this study, a maximal abundance of 4% was found for this phylum. Although this value is at the lower end of the observed range, the abundance is still remarkable given that a relative abundance of 0.03% was observed for the phylum in samples from the same geographical location and with a similar temperature profile (67). Most often, members of the families *Planctomycetaceae* and *Pirellulaceae* appeared in the free-living fraction obtained from the transition zone. This is not surprising as most of the characterized species of these families are mesophiles showing optimal growth at temperatures of 26°C–30°C. Although the temperature optimum nicely reflects the temperature of 27.6°C found at the sampling location, this does not exclude that species from other planctomycetal families might be present, for example, members of the family *Thermoguttaceae*. Species belonging to this family are thermophilic and capable of growth under anaerobic conditions using nitrate or sulfur compounds as electron acceptors (64). The type strain of one of these species, *Thermostilla marina*, was isolated from Vulcano Island, which is located only 25 km away from Panarea (56). Strain Pan216^T^ was isolated from a gelatinous hemisphere. These gelatinous textures, which are associated with hydrothermal vents, can be heavily encrusted with rust-colored Fe oxides and are often colonized by iron- and sulfur-oxidizing bacteria (69). Within this environment, hydrothermal vents or iron-hydroxide deposits can be a source of novel phylum members as previously demonstrated (70, 71).

### Cell division by lateral budding

Besides its phylogenetic distance as a member of a novel family, strain Pan216^T^ also showed uncommon cell biological features. Cell division by lateral budding is a hallmark feature of the strain. A related mode of cell division has so far only been observed in two planctomycetal strains belonging to the provisional species “*Candidatus* Laterigemmans baculatus” (72). Unfortunately, both strains lost viability on passaging and are no longer available for more detailed analyses. The two strains were shown to be members of the family *Pirellulaceae,* and strain Pan216^T^ is hence not their closest relative.

Lateral budding in strain Pan216^T^ is initiated by a reduction of the circumference of the midcell, leading to bending of the cell, yielding a more kidney-like shape of the mother cell. The bud originates from the midcell perpendicular to the longitudinal axis without any apparent involvement of the cell poles. Since the divisome of “regularly” polar budding-planctomycetes is not understood, speculation on the exceptional mode of cell division in strain Pan216^T^ appears even more challenging. Models for the bacterial cell division cycle have been based largely on either species exhibiting symmetric binary fission or on *Caulobacter* spp., which displays asymmetric division correlated with a prosthecate stalk (73). The genome-based analysis of putative divisome genes did not yield significant differences when comparing this strain with representative members of planctomycetal families that perform different modes of cell division (23). All known planctomycetes (including the ones dividing by binary fission) lack the otherwise universal Z ring-forming FtsZ (11). The genes encoding proteins FtsI, FtsW, and MreB were shown to be non-essential in *P. limnophila*. This said, it should be considered that planctomycetes use an uncharacterized or evolutionary distinct set of proteins during cell division. Although no transitional form between budding and binary fission has been found so far, evolutionary transition from binary fission to budding is discussed (23). Comparative genomic analyses of strains following these different types of cell division might reveal genes that are involved in the cell division or in the positioning of the cell division machinery, which ultimately also help to decipher the specific cell division mechanism of planctomycetes.

### S-layer formation

An S-layer was identified on the cell surface of strain Pan216^T^. In Gram-negative bacteria, the S-layer is linked to a smooth lipopolysaccharide (LPS) which is anchored into the outer membrane (74), while in archaea, the S-layer relates to the cytoplasmic membrane (75). Previous proteomic studies have shown that S-layer proteins are usually among the 20 highest abundance proteins (76, 77). However, S-layer protein-encoding genes are difficult to identify using *in silico* analyses as they are typically not homologous between different bacterial species (78). A BLASTp analysis revealed that homologs of the kustd1514-encoded protein cannot be found in any other bacterium, (77) including Pan216^T^. Similar to the mode of cell division, the nature of the S-layer in Pan216^T^ requires experimental effort in follow-up studies based on the identified candidate genes.

### Conclusion

Our numerous analyses, including phylogenetic, physiological, chemotaxonomic, and comparative genomics, yielded many differences between the novel isolate and members of the described families in different orders of the class *Planctomycetia*. Taken together, our results justify the separation of the novel isolate from the described families in the mentioned class. Thus, we introduce the species *Kolteria novifilia* gen. nov., sp. nov. with Pan216^T^ as the type strain along with *Kolteriaceae* fam. nov. and *Kolteriales* ord. nov.

### Description of *Kolteria* gen. nov

*Kolteria* (Kol.te’ri.a. N.L. fem. n. *Kolteria* of Kolter, a bacterium named in honor of Roberto Kolter. Roberto has a far-reaching impact on the field of microbiology through his outstanding scientific contributions, his exceptional presentation and teaching skills, and the mentorship he provided to so many scientists around the globe).

The oblong-shaped cells form no stable aggregates or rosettes under aerobic culture conditions. Daughter cells are highly motile, while mother cells are non-motile and non-stalk forming. The cell plan is Gram-negative. The surface is rough and surrounded by an additional outer surface layer. No crateriform structures could be observed. Cells reproduce by lateral budding while mother and daughter cells are connected by a thin tubular-like structure. Cells are heterotrophic, facultatively anaerobic, and mesophilic. The type species is *Kolteria novifilia*.

### Description of *Kolteria novifilia* sp. nov

*Kolteria novifilia* (no.vi.fi’li.a. L. fem. adj. *nova* new; L. fem. n. *filia* a daughter; N.L. fem. n. *novifilia*; corresponding to the ability of the cells to generate daughter cells).

In addition to the features described for the genus, the species exhibits the following properties. Colonies grown on M1H NAG ASW agar were round, smooth, and unpigmented. Cells are approximately 2.1 µm in length and 1.3 µm in width. Non-motile mother cells spawn motile, swarming daughter cells. Oxidase and catalase activities were positive. Cells used the following carbon sources for biomass formation: adonitol, fermented rumen extract, gluconate, lactate, maltose, mannitol, melizitose, *N*-acetyl galactosamine, *N*-acetyl glucosamine, ornithine, protocatechuate, raffinose, shikimate, sucrose, trehalose, xylose, and yeast extract. The APIZYM test was positive for leucine arylamidase. Major fatty acids are 14:0, 16:0, and 18:1 ω7c and summed feature 16:1 ω7c/15 iso 2-OH. The DNA G + C content is around 62.0%. Growth occurs between pH 6.0 and 10.0 with an optimum at pH 8.0. At least 10% ASW is needed for growth, and up to 100% is tolerated. NaCl concentrations from 4% to 7% (wt/vol) allowed for the growth of the strain. The optimal temperature for growth is 32°C (range between 16°C and 34°C). The type strain is Pan216^T^ (= DSM 100414^T^ = CECT 9536^T^) isolated from a gelatinous hemisphere found in the hydrothermal vent system close to Panarea Island, Italy.

### Description of *Kolteriaceae* fam. nov

*Kolteriaceae* (Kol.te’ri.a.ce’ae. N.L. fem. n, *Kolteria* type genus of the family; suffix. *-aceae* ending to denote a family; N.L. fem. pl. n. *Kolteriaceae*, the *Kolteria* family). The type genus of the family is *Kolteria*.

### Description of *Kolteriales* ord. nov

*Kolteriales* (Kol.te’ri.a.les. N.L. masc. dim. n. *Kolteria*, type genus of the order; suff. -*ales*, ending to denote an order; N.L. fem. pl. n. *Kolteriales*, the *Kolteria* order). The type genus of the order is *Kolteria*.

## Data Availability

GenBank accession numbers for the genome and 16S rRNA gene sequence of the novel isolate, as well as microbial strain collection deposition numbers for the type strain, are provided in the manuscript text.

## SUPPLEMENTAL MATERIAL

Supplemental figures and tables jb.00337-24-s0001.pdf Figures S1 to S3 and Tables S1 to S10.

Figure S1 jb.00337-24-s0002.pdf 16S Phylogenetic tree (amplicon sequences).

Table S1 jb.00337-24-s0003.xlsx Operational taxonomic units obtained from the attached fraction of surface watter.

Table S2 jb.00337-24-s0004.xlsx Species assignment for unique sequences belonging to the phylum *Planctomycetota*.

Table S5 jb.00337-24-s0005.xlsx Core genome analysis based on the comparison of strain Pan216 with either non-budding-, budding- or all sequenced *planctomycetes*.

Table S7 jb.00337-24-s0006.xlsx Putative S-layer proteins in strain Pan216.

Movie S1 jb.00337-24-s0007.mpg Cell division of *Planctopirus limnophila*.

Movie S2 jb.00337-24-s0008.mpg Cell division of *Gemmata obscuriglobus*.

Movie S3 jb.00337-24-s0009.mpg Cell division of strain Pan216^T^.

ASM does not own the copyrights to Supplemental Material that may be linked to, or accessed through, an article. The authors have granted ASM a non-exclusive, world-wide license to publish the Supplemental Material files. Please contact the corresponding author directly for reuse.

## References

[1] Fuerst JA, Sagulenko E. 2011. Beyond the bacterium: planctomycetes challenge our concepts of microbial structure and function. Nat Rev Microbiol 9:403–413. doi:10.1038/nrmicro2578 21572457

[2] Kartal B, de Almeida NM, Maalcke WJ, Op den Camp HJM, Jetten MSM, Keltjens JT. 2013. How to make a living from anaerobic ammonium oxidation. FEMS Microbiol Rev 37:428–461. doi:10.1111/1574-6976.12014 23210799

[3] Wagner M, Horn M. 2006. The Planctomycetes, Verrucomicrobia, Chlamydiae and sister phyla comprise a superphylum with biotechnological and medical relevance. Curr Opin Biotechnol 17:241–249. doi:10.1016/j.copbio.2006.05.005 16704931

[4] Spring S, Bunk B, Spröer C, Schumann P, Rohde M, Tindall BJ, Klenk H-P. 2016. Characterization of the first cultured representative of Verrucomicrobia subdivision 5 indicates the proposal of a novel phylum. ISME J 10:2801–2816. doi:10.1038/ismej.2016.84 27300277 PMC5148204

[5] Kallscheuer N, Wurzbacher CE, Schmitz RA, Jogler C. 2024. In the footsteps of Heinz Schlesner and Peter Hirsch: exploring the untapped diversity of the phylum Planctomycetota in isolates from the 1980s to the early 2000s. Syst Appl Microbiol 47:126486. doi:10.1016/j.syapm.2023.126486 38104493

[6] Wiegand S, Jogler M, Jogler C. 2018. On the maverick Planctomycetes. FEMS Microbiol Rev 42:739–760. doi:10.1093/femsre/fuy029 30052954

[7] Lage OM, Bondoso J. 2014. Planctomycetes and macroalgae, a striking association. Front Microbiol 5:267. doi:10.3389/fmicb.2014.00267 24917860 PMC4042473

[8] Kohn T, Rast P, Kallscheuer N, Wiegand S, Boedeker C, Jetten MSM, Jeske O, Vollmers J, Kaster A-K, Rohde M, Jogler M, Jogler C. 2020. The microbiome of Posidonia oceanica seagrass leaves can be dominated by Planctomycetes. Front Microbiol 11:1458. doi:10.3389/fmicb.2020.01458 32754127 PMC7366357

[9] Storesund JE, Lanzèn A, García-Moyano A, Reysenbach A-L, Øvreås L. 2018. Diversity patterns and isolation of Planctomycetes associated with metalliferous deposits from hydrothermal vent fields along the Valu Fa Ridge (SW Pacific). Antonie Van Leeuwenhoek 111:841–858. doi:10.1007/s10482-018-1026-8 29423768

[10] Bengtsson MM, Øvreås L. 2010. Planctomycetes dominate biofilms on surfaces of the kelp Laminaria hyperborea. BMC Microbiol 10:261. doi:10.1186/1471-2180-10-261 20950420 PMC2964680

[11] Wiegand S, Jogler M, Boedeker C, Pinto D, Vollmers J, Rivas-Marín E, Kohn T, Peeters SH, Heuer A, Rast P, et al.. 2020. Cultivation and functional characterization of 79 planctomycetes uncovers their unique biology. Nat Microbiol 5:126–140. doi:10.1038/s41564-019-0588-1 31740763 PMC7286433

[12] Kallscheuer N, Jogler C. 2021. The bacterial phylum Planctomycetes as novel source for bioactive small molecules. Biotechnol Adv 53:107818. doi:10.1016/j.biotechadv.2021.107818 34537319

[13] Vitorino IR, Lage OM. 2022. The Planctomycetia: an overview of the currently largest class within the phylum Planctomycetes. Antonie Van Leeuwenhoek 115:169–201. doi:10.1007/s10482-021-01699-0 35037113

[14] Ward NL. 2010. Phylum XXV. Planctomycetes Garrity and Holt 2001, 137 emend. Ward (this volume), p 879–925. In Krieg NR, Staley JT, Brown DR, Hedlund BP, Paster BJ, Ward NL, Ludwig W, Whitman WB (ed), Bergey’s manual of systematic bacteriology: volume four the Bacteroidetes, Spirochaetes, Tenericutes (Mollicutes), Acidobacteria, Fibrobacteres, Fusobacteria, Dictyoglomi, Gemmatimonadetes, Lentisphaerae, Verrucomicrobia, Chlamydiae, and Planctomycetes. Springer, New York, NY.doi:10.1007/978-0-387-68572-4_14

[15] Fukunaga Y, Kurahashi M, Sakiyama Y, Ohuchi M, Yokota A, Harayama S. 2009. Phycisphaera mikurensis gen. nov., sp. nov., isolated from a marine alga, and proposal of Phycisphaeraceae fam. nov., Phycisphaerales ord. nov. and Phycisphaerae classis nov. in the phylum Planctomycetes. J Gen Appl Microbiol 55:267–275. doi:10.2323/jgam.55.267 19700920

[16] Jenkins C, Staley JT. 2013. History, classification and cultivation of the planctomycetes. In Fuerst JA (ed), Planctomycetes: cell structure, origins and biology. Humana Press, Totowa, NJ.

[17] Lodha T, Narvekar S, Karodi P. 2021. Classification of uncultivated anammox bacteria and Candidatus Uabimicrobium into new classes and provisional nomenclature as Candidatus Brocadiia classis nov. and Candidatus Uabimicrobiia classis nov. of the phylum Planctomycetes and novel family Candidatus Scalinduaceae fam. nov to accommodate the genus Candidatus Scalindua. Syst Appl Microbiol 44:126272. doi:10.1016/j.syapm.2021.126272 34735804

[18] Rivas-Marín E, Devos DP. 2018. The paradigms they are a-Changin': past, present and future of PVC bacteria research. Antonie Van Leeuwenhoek 111:785–799. doi:10.1007/s10482-017-0962-z 29058138 PMC5945725

[19] Rast P, Glöckner I, Boedeker C, Jeske O, Wiegand S, Reinhardt R, Schumann P, Rohde M, Spring S, Glöckner FO, Jogler C, Jogler M. 2017. Three novel species with peptidoglycan cell walls form the new genus Lacunisphaera gen. nov. in the family Opitutaceae of the verrucomicrobial subdivision 4. Front Microbiol 8:202. doi:10.3389/fmicb.2017.00202 28243229 PMC5303756

[20] Hirsch P, Müller M. 1985. Planctomyces limnophilus sp. nov., a stalked and budding bacterium from freshwater. Syst Appl Microbiol 6:276–280. doi:10.1016/S0723-2020(85)80031-X

[21] Lee KC, Webb RI, Fuerst JA. 2009. The cell cycle of the planctomycete Gemmata obscuriglobus with respect to cell compartmentalization. BMC Cell Biol 10:4. doi:10.1186/1471-2121-10-4 19144151 PMC2656463

[22] Kovaleva OL, Merkel AY, Novikov AA, Baslerov RV, Toshchakov SV, Bonch-Osmolovskaya EA. 2015. Tepidisphaera mucosa gen. nov., sp. nov., a moderately thermophilic member of the class Phycisphaerae in the phylum Planctomycetes, and proposal of a new family, Tepidisphaeraceae fam. nov., and a new order, Tepidisphaerales ord. nov. Int J Syst Evol Microbiol 65:549–555. doi:10.1099/ijs.0.070151-0 25404483

[23] Rivas-Marín E, Canosa I, Devos DP. 2016. Evolutionary cell biology of division mode in the bacterial Planctomycetes-Verrucomicrobia-Chlamydiae superphylum. Front Microbiol 7:1964. doi:10.3389/fmicb.2016.01964 28018303 PMC5147048

[24] van Niftrik L, Geerts WJC, van Donselaar EG, Humbel BM, Webb RI, Harhangi HR, Camp HJMO den, Fuerst JA, Verkleij AJ, Jetten MSM, Strous M. 2009. Cell division ring, a new cell division protein and vertical inheritance of a bacterial organelle in anammox planctomycetes. Mol Microbiol 73:1009–1019. doi:10.1111/j.1365-2958.2009.06841.x 19708922

[25] Jogler C, Glöckner FO, Kolter R. 2011. Characterization of Planctomyces limnophilus and development of genetic tools for its manipulation establish it as a model species for the phylum Planctomycetes. Appl Environ Microbiol 77:5826–5829. doi:10.1128/AEM.05132-11 21724885 PMC3165242

[26] Wecker P, Klockow C, Schüler M, Dabin J, Michel G, Glöckner FO. 2010. Life cycle analysis of the model organism Rhodopirellula baltica SH 1^T^ by transcriptome studies. Microb Biotechnol 3:583–594. doi:10.1111/j.1751-7915.2010.00183.x 21255355 PMC3815771

[27] Jogler C, Waldmann J, Huang X, Jogler M, Glöckner FO, Mascher T, Kolter R. 2012. Identification of proteins likely to be involved in morphogenesis, cell division, and signal transduction in Planctomycetes by comparative genomics. J Bacteriol 194:6419–6430. doi:10.1128/JB.01325-12 23002222 PMC3497475

[28] Pilhofer M, Rappl K, Eckl C, Bauer AP, Ludwig W, Schleifer KH, Petroni G. 2008. Characterization and evolution of cell division and cell wall synthesis genes in the bacterial phyla Verrucomicrobia, Lentisphaerae, Chlamydiae, and Planctomycetes and phylogenetic comparison with rRNA genes. J Bacteriol 190:3192–3202. doi:10.1128/JB.01797-07 18310338 PMC2347405

[29] Price RE, LaRowe DE, Italiano F, Savov IP, Pichler T, Amend JP. 2017. Geochemical analyses in hydrothermal fluids from three shallow-sea vents offshore Panarea. PANGAEA. doi:10.1594/PANGAEA.873672

[30] Blanco L, Bernad A, Lázaro JM, Martín G, Garmendia C, Salas M. 1989. Highly efficient DNA synthesis by the phage ϕ 29 DNA polymerase: symmetrical mode of DNA replication. J Biol Chem 264:8935–8940. doi:10.1016/S0021-9258(18)81883-X 2498321

[31] Muyzer G, de Waal EC, Uitterlinden AG. 1993. Profiling of complex microbial populations by denaturing gradient gel electrophoresis analysis of polymerase chain reaction-amplified genes coding for 16S rRNA. Appl Environ Microbiol 59:695–700. doi:10.1128/aem.59.3.695-700.1993 7683183 PMC202176

[32] Bartram AK, Lynch MDJ, Stearns JC, Moreno-Hagelsieb G, Neufeld JD. 2011. Generation of multimillion-sequence 16S rRNA gene libraries from complex microbial communities by assembling paired-end illumina reads. Appl Environ Microbiol 77:3846–3852. doi:10.1128/AEM.02772-10 21460107 PMC3127616

[33] Rognes T, Flouri T, Nichols B, Quince C, Mahé F. 2016. VSEARCH: a versatile open source tool for metagenomics. PeerJ 4:e2584. doi:10.7717/peerj.2584 27781170 PMC5075697

[34] Bokulich NA, Subramanian S, Faith JJ, Gevers D, Gordon JI, Knight R, Mills DA, Caporaso JG. 2013. Quality-filtering vastly improves diversity estimates from Illumina amplicon sequencing. Nat Methods 10:57–59. doi:10.1038/nmeth.2276 23202435 PMC3531572

[35] Amir A, McDonald D, Navas-Molina JA, Kopylova E, Morton JT, Zech Xu Z, Kightley EP, Thompson LR, Hyde ER, Gonzalez A, Knight R. 2017. Deblur rapidly resolves single-nucleotide community sequence patterns. mSystems 2:e00191-16. doi:10.1128/mSystems.00191-16 28289731 PMC5340863

[36] Quast C, Pruesse E, Yilmaz P, Gerken J, Schweer T, Yarza P, Peplies J, Glöckner FO. 2013. The SILVA ribosomal RNA gene database project: improved data processing and web-based tools. Nucleic Acids Res 41:D590–D596. doi:10.1093/nar/gks1219 23193283 PMC3531112

[37] Strous M, Heijnen JJ, Kuenen JG, Jetten MSM. 1998. The sequencing batch reactor as a powerful tool for the study of slowly growing anaerobic ammonium-oxidizing microorganisms. Appl Microbiol Biotechnol 50:589–596. doi:10.1007/s002530051340

[38] Jeske O, Surup F, Ketteniß M, Rast P, Förster B, Jogler M, Wink J, Jogler C. 2016. Developing techniques for the utilization of Planctomycetes as producers of bioactive molecules. Front Microbiol 7:1242. doi:10.3389/fmicb.2016.01242 27594849 PMC4990742

[39] Wurzbacher CE, Haufschild T, Hammer J, van Teeseling MCF, Kallscheuer N, Jogler C. 2024. Planctoellipticum variicoloris gen. nov., sp. nov., a novel member of the family Planctomycetaceae isolated from wastewater of the aeration lagoon of a sugar processing plant in Northern Germany. Sci Rep 14:5741. doi:10.1038/s41598-024-56373-y 38459238 PMC10923784

[40] Rodriguez-R LM, Konstantinidis KT. 2016. The enveomics collection: a toolbox for specialized analyses of microbial genomes and metagenomes. PeerJ Preprints. doi:10.7287/peerj.preprints.1900v1

[41] Qin QL, Xie BB, Zhang XY, Chen XL, Zhou BC, Zhou J, Oren A, Zhang YZ. 2014. A proposed genus boundary for the prokaryotes based on genomic insights. J Bacteriol 196:2210–2215. doi:10.1128/JB.01688-14 24706738 PMC4054180

[42] Bondoso J, Harder J, Lage OM. 2013. rpoB gene as a novel molecular marker to infer phylogeny in Planctomycetales. Antonie Van Leeuwenhoek 104:477–488. doi:10.1007/s10482-013-9980-7 23904187

[43] Pascual J, Foesel BU, Geppert A, Huber KJ, Boedeker C, Luckner M, Wanner G, Overmann J. 2018. Roseisolibacter agri gen. nov., sp. nov., a novel slow-growing member of the under-represented phylum Gemmatimonadetes. Int J Syst Evol Microbiol 68:1028–1036. doi:10.1099/ijsem.0.002619 29458671

[44] Wittmann J, Dreiseikelmann B, Rohde C, Rohde M, Sikorski J. 2014. Isolation and characterization of numerous novel phages targeting diverse strains of the ubiquitous and opportunistic pathogen Achromobacter xylosoxidans. PLoS One 9:e86935. doi:10.1371/journal.pone.0086935 24466294 PMC3899368

[45] Kohn T, Heuer A, Jogler M, Vollmers J, Boedeker C, Bunk B, Rast P, Borchert D, Glöckner I, Freese HM, Klenk H-P, Overmann J, Kaster A-K, Rohde M, Wiegand S, Jogler C. 2016. Fuerstia marisgermanicae gen. nov., sp. nov., an unusual member of the phylum Planctomycetes from the German Wadden Sea. Front Microbiol 7:2079. doi:10.3389/fmicb.2016.02079 28066393 PMC5177795

[46] Huber KJ, Wüst PK, Rohde M, Overmann J, Foesel BU. 2014. Aridibacter famidurans gen. nov., sp. nov. and Aridibacter kavangonensis sp. nov., two novel members of subdivision 4 of the Acidobacteria isolated from semiarid savannah soil. Int J Syst Evol Microbiol 64:1866–1875. doi:10.1099/ijs.0.060236-0 24573163

[47] Sasser M. 1990. Identification of bacteria by gas chromatography of cellular fatty acids methyl esters(GC-FAME). Tech note # 101 Internal MIDI document revised 2006

[48] Lechner M, Findeiss S, Steiner L, Marz M, Stadler PF, Prohaska SJ. 2011. Proteinortho: detection of (co-)orthologs in large-scale analysis. BMC Bioinformatics 12:124. doi:10.1186/1471-2105-12-124 21526987 PMC3114741

[49] Huerta-Cepas J, Szklarczyk D, Forslund K, Cook H, Heller D, Walter MC, Rattei T, Mende DR, Sunagawa S, Kuhn M, Jensen LJ, von Mering C, Bork P. 2016. eggNOG 4.5: a hierarchical orthology framework with improved functional annotations for eukaryotic, prokaryotic and viral sequences. Nucleic Acids Res 44:D286–D293. doi:10.1093/nar/gkv1248 26582926 PMC4702882

[50] Bertelli C, Laird MR, Williams KP, Lau BY, Hoad G, Winsor GL, Brinkman FSL, Simon Fraser University Research Computing Group. 2017. IslandViewer 4: expanded prediction of genomic islands for larger-scale datasets. Nucleic Acids Res 45:W30–W35. doi:10.1093/nar/gkx343 28472413 PMC5570257

[51] Alikhan N-F, Petty NK, Ben Zakour NL, Beatson SA. 2011. BLAST Ring Image Generator (BRIG): simple prokaryote genome comparisons. BMC Genomics 12:402. doi:10.1186/1471-2164-12-402 21824423 PMC3163573

[52] Fagan RP, Fairweather NF. 2014. Biogenesis and functions of bacterial S-layers. Nat Rev Microbiol 12:211–222. doi:10.1038/nrmicro3213 24509785

[53] Artimo P, Jonnalagedda M, Arnold K, Baratin D, Csardi G, de Castro E, Duvaud S, Flegel V, Fortier A, Gasteiger E, Grosdidier A, Hernandez C, Ioannidis V, Kuznetsov D, Liechti R, Moretti S, Mostaguir K, Redaschi N, Rossier G, Xenarios I, Stockinger H. 2012. ExPASy: SIB bioinformatics resource portal. Nucleic Acids Res 40:W597–W603. doi:10.1093/nar/gks400 22661580 PMC3394269

[54] Hunter S, Apweiler R, Attwood TK, Bairoch A, Bateman A, Binns D, Bork P, Das U, Daugherty L, Duquenne L, et al.. 2009. InterPro: the integrative protein signature database. Nucleic Acids Res 37:D211–D215. doi:10.1093/nar/gkn785 18940856 PMC2686546

[55] Oren A, Garrity GM. 2021. Valid publication of the names of forty-two phyla of prokaryotes. Int J Syst Evol Microbiol 71:005056. doi:10.1099/ijsem.0.005056 34694987

[56] Slobodkina GB, Panteleeva AN, Beskorovaynaya DA, Bonch-Osmolovskaya EA, Slobodkin AI. 2016. Thermostilla marina gen. nov., sp. nov., a thermophilic, facultatively anaerobic planctomycete isolated from a shallow submarine hydrothermal vent. Int J Syst Evol Microbiol 66:633–638. doi:10.1099/ijsem.0.000767 26559645

[57] Wiegand S, Jogler M, Kohn T, Awal RP, Oberbeckmann S, Kesy K, Jeske O, Schumann P, Peeters SH, Kallscheuer N, Strauss M, Heuer A, Jetten MSM, Labrenz M, Rohde M, Boedeker C, Engelhardt H, Schüler D, Jogler C. 2019. The novel shapeshifting bacterial phylum Saltatorellota. bioRxiv. doi:10.1101/817700

[58] Kallscheuer N, Jogler M, Wiegand S, Peeters SH, Heuer A, Boedeker C, Jetten MSM, Rohde M, Jogler C. 2020. Rubinisphaera italica sp. nov. isolated from a hydrothermal area in the Tyrrhenian Sea close to the volcanic island Panarea. Antonie Van Leeuwenhoek 113:1727–1736. doi:10.1007/s10482-019-01329-w 31773447 PMC7717053

[59] Rensink S, Wiegand S, Kallscheuer N, Rast P, Peeters SH, Heuer A, Boedeker C, Jetten MSM, Rohde M, Jogler M, Jogler C. 2020. Description of the novel planctomycetal genus Bremerella, containing Bremerella volcania sp. nov., isolated from an active volcanic site, and reclassification of Blastopirellula cremea as Bremerella cremea comb. nov. Antonie Van Leeuwenhoek 113:1823–1837. doi:10.1007/s10482-019-01378-1 31894496

[60] Kumar G, Kallscheuer N, Jogler M, Wiegand S, Heuer A, Boedeker C, Rohde M, Jogler C. 2023. Stratiformator vulcanicus gen. nov., sp. nov., a marine member of the family Planctomycetaceae isolated from a red biofilm in the Tyrrhenian Sea close to the volcanic island Panarea. Antonie Van Leeuwenhoek 116:995–1007. doi:10.1007/s10482-023-01860-x 37584762 PMC10509075

[61] Yarza P, Yilmaz P, Pruesse E, Glöckner FO, Ludwig W, Schleifer K-H, Whitman WB, Euzéby J, Amann R, Rosselló-Móra R. 2014. Uniting the classification of cultured and uncultured bacteria and archaea using 16S rRNA gene sequences. Nat Rev Microbiol 12:635–645. doi:10.1038/nrmicro3330 25118885

[62] Konstantinidis KT, Tiedje JM. 2005. Towards a genome-based taxonomy for prokaryotes. J Bacteriol 187:6258–6264. doi:10.1128/JB.187.18.6258-6264.2005 16159757 PMC1236649

[63] Kallscheuer N, Wiegand S, Peeters SH, Jogler M, Boedeker C, Heuer A, Rast P, Jetten MSM, Rohde M, Jogler C. 2020. Description of three bacterial strains belonging to the new genus Novipirellula gen. nov., reclassificiation of Rhodopirellula rosea and Rhodopirellula caenicola and readjustment of the genus threshold of the phylogenetic marker rpoB for Planctomycetaceae. Antonie Van Leeuwenhoek 113:1779–1795. doi:10.1007/s10482-019-01374-5 31853689

[64] Dedysh SN, Kulichevskaya IS, Beletsky AV, Ivanova AA, Rijpstra WIC, Damsté JSS, Mardanov AV, Ravin NV. 2020. Lacipirellula parvula gen. nov., sp. nov., representing a lineage of planctomycetes widespread in low-oxygen habitats, description of the family Lacipirellulaceae fam. nov. and proposal of the orders Pirellulales ord. nov., Gemmatales ord. nov. and Isosphaerales ord. nov. Syst Appl Microbiol 43:126050. doi:10.1016/j.syapm.2019.126050 31882205 PMC6995999

[65] Bondoso J, Albuquerque L, Nobre MF, Lobo-da-Cunha A, da Costa MS, Lage OM. 2011. Aquisphaera giovannonii gen. nov., sp. nov., a planctomycete isolated from a freshwater aquarium. Int J Syst Evol Microbiol 61:2844–2850. doi:10.1099/ijs.0.027474-0 21239565

[66] van Teeseling MCF, Maresch D, Rath CB, Figl R, Altmann F, Jetten MSM, Messner P, Schäffer C, van Niftrik L. 2016. The S-layer protein of the anammox bacterium Kuenenia stuttgartiensis is heavily O-glycosylated. Front Microbiol 7:1721. doi:10.3389/fmicb.2016.01721 27847504 PMC5088730

[67] Gugliandolo C, Lentini V, Bunk B, Overmann J, Italiano F, Maugeri TL. 2015. Changes in prokaryotic community composition accompanying a pronounced temperature shift of a shallow marine thermal brine pool (Panarea Island, Italy). Extremophiles 19:547–559. doi:10.1007/s00792-015-0737-2 25716144

[68] Cai H-Y, Yan Z, Wang A-J, Krumholz LR, Jiang H-L. 2013. Analysis of the attached microbial community on mucilaginous cyanobacterial aggregates in the eutrophic Lake Taihu reveals the importance of Planctomycetes. Microb Ecol 66:73–83. doi:10.1007/s00248-013-0224-1 23571665

[69] Emerson D, Moyer CL. 2002. Neutrophilic Fe-oxidizing bacteria are abundant at the Loihi Seamount hydrothermal vents and play a major role in Fe oxide deposition. Appl Environ Microbiol 68:3085–3093. doi:10.1128/AEM.68.6.3085-3093.2002 12039770 PMC123976

[70] Storesund JE, Øvreås L. 2013. Diversity of Planctomycetes in iron-hydroxide deposits from the Arctic Mid Ocean Ridge (AMOR) and description of Bythopirellula goksoyri gen. nov., sp. nov., a novel Planctomycete from deep sea iron-hydroxide deposits. Antonie Van Leeuwenhoek 104:569–584. doi:10.1007/s10482-013-0019-x 24018702

[71] Slobodkina GB, Kovaleva OL, Miroshnichenko ML, Slobodkin AI, Kolganova TV, Novikov AA, van Heerden E, Bonch-Osmolovskaya EA. 2015. Thermogutta terrifontis gen. nov., sp. nov. and Thermogutta hypogea sp. nov., thermophilic anaerobic representatives of the phylum Planctomycetes. Int J Syst Evol Microbiol 65:760–765. doi:10.1099/ijs.0.000009 25479950

[72] Kumar D, Kumar G, Jagadeeshwari U, Sasikala C, Ramana CV. 2021. “Candidatus Laterigemmans baculatus” gen. nov. sp. nov., the first representative of rod shaped planctomycetes with lateral budding in the family Pirellulaceae. Syst Appl Microbiol 44:126188. doi:10.1016/j.syapm.2021.126188 33647766

[73] Angert ER. 2005. Alternatives to binary fission in bacteria. Nat Rev Microbiol 3:214–224. doi:10.1038/nrmicro1096 15738949

[74] Boot HJ, Pouwels PH. 1996. Expression, secretion and antigenic variation of bacterial S-layer proteins. Mol Microbiol 21:1117–1123. doi:10.1046/j.1365-2958.1996.711442.x 8898381

[75] Haft DH, Payne SH, Selengut JD. 2012. Archaeosortases and exosortases are widely distributed systems linking membrane transit with posttranslational modification. J Bacteriol 194:36–48. doi:10.1128/JB.06026-11 22037399 PMC3256604

[76] Chiou SY, Kang PL, Liao TW, Jeang CL. 2008. Characterization, identification, and cloning of the S-layer protein from Cytophaga sp. Curr Microbiol 56:597–602. doi:10.1007/s00284-008-9132-x 18322733

[77] van Teeseling MCF, de Almeida NM, Klingl A, Speth DR, Op den Camp HJM, Rachel R, Jetten MSM, van Niftrik L. 2014. A new addition to the cell plan of anammox bacteria: “Candidatus Kuenenia stuttgartiensis” has a protein surface layer as the outermost layer of the cell. J Bacteriol 196:80–89. doi:10.1128/JB.00988-13 24142254 PMC3911120

[78] Engelhardt H, Peters J. 1998. Structural research on surface layers: a focus on stability, surface layer homology domains, and surface layer-cell wall interactions. J Struct Biol 124:276–302. doi:10.1006/jsbi.1998.4070 10049812

