# Supplementary material for: Kolteria novifilia, a novel planctomycetotal strain from the volcanic habitat of Panarea divides by unusual lateral budding
Source: J Bacteriol. 2025 Jun 24;207(7):e00337-24. doi: 10.1128/jb.00337-24 (PMC12288469; doi:10.1128/jb.00337-24)
Supplement: Supplemental figures and tables — Figures S1 to S3 and Tables S1 to S10. [file jb.00337-24-s0001.pdf]

Supporting Information

Kolteria novifilia, **a novel planctomycetotal strain from the volcanic habitat of Panarea divides by unusual lateral budding**

Nicolai Kallscheuer[1,#], Christian Boedeker[2,#], Sandra Wiegand[3,4], Timo Kohn[4], Anja Heuer[2], Jörg Overmann[2,5], Stijn Peters[4], Mareike Jogler[1], Manfred Rohde[6] and Christian Jogler[1,7,*]

[1] Department of Microbial Interactions, Friedrich Schiller-University, Jena, Germany

[2] Leibniz Institute DSMZ-German Collection of Microorganisms and Cell Cultures, Braunschweig, Germany

[3] Institute for Biological Interfaces 5, Karlsruhe Institute of Technology, Eggenstein-Leopoldshafen, Germany

[4] Radboud University, Department of Microbiology, Nijmegen, The Netherlands

[5] Microbiology, Technical University of Braunschweig, Braunschweig, Germany

[6] Helmholtz Centre for Infection Research, Central Facility for Microscopy, Braunschweig, Germany

[7] Cluster of Excellence Balance of the Microverse, Friedrich Schiller University, Jena, Germany

# authors contributed equally

***Correspondence:** Christian Jogler; christian.jogler@uni-jena.de

Running title: uncommon planctomycetal cell division by lateral budding

Keywords: Planctomycetes, cell division, budding, binary fission, surface layer, *Panarea*, 16S rRNA, V3 region

Supplementary Figures

Figure S1. Phylogenetic tree based on planctomycetal 16S rRNA gene sequences from the amplicon data set and of described members of the phylum (as of June 2024).

Figure 1 is provided as separate PDF file.

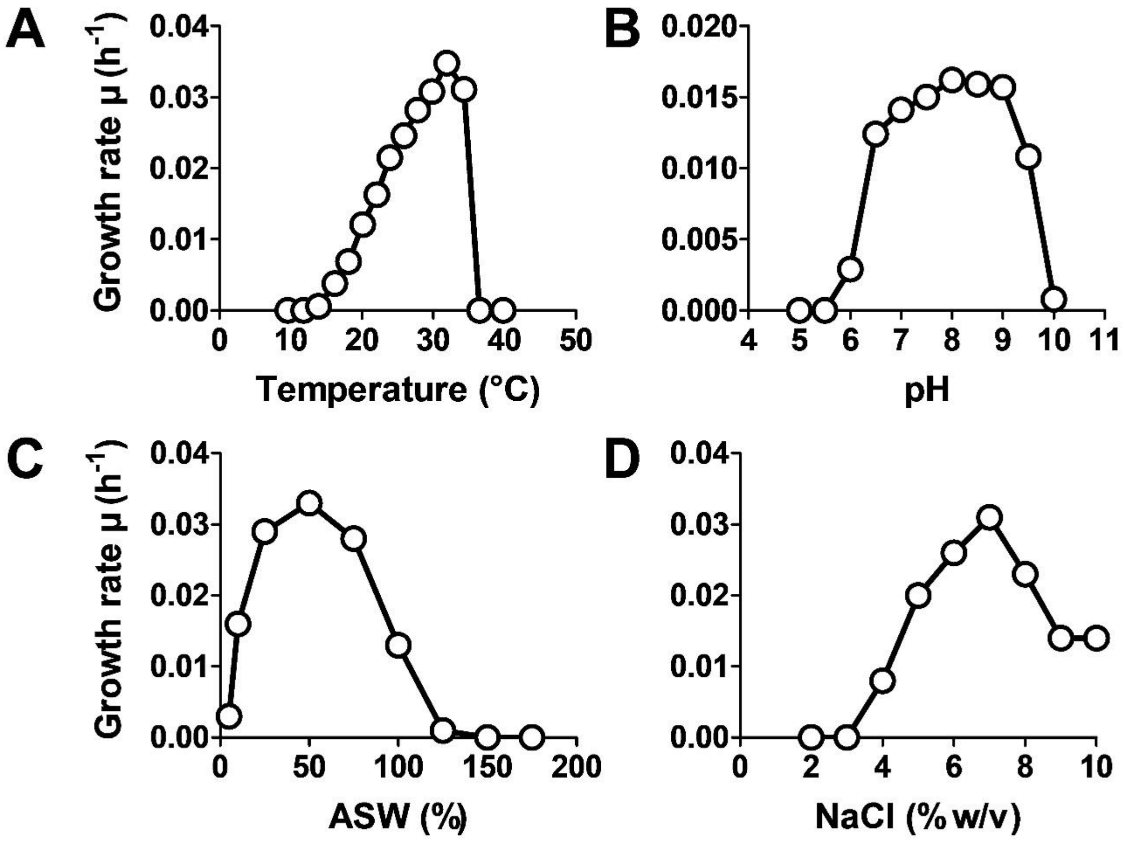

Figure S2. Influence of temperature, pH, artificial seawater (ASW) and NaCl concentration on growth of strain Pan216^T. To determine the optimum growth temperature (A), pH (B), ASW concentration (C) and NaCl concentration (D) cell growth was measured as optical density at 600 nm (OD_{600}). Temperature, pH, ASW and NaCl optima of strain Pan216^T were determined to be 32 °C, pH 8.0, 50% ASW and 7% (w/v) NaCl. Each dot represents the average growth rate of triplicate measurements.

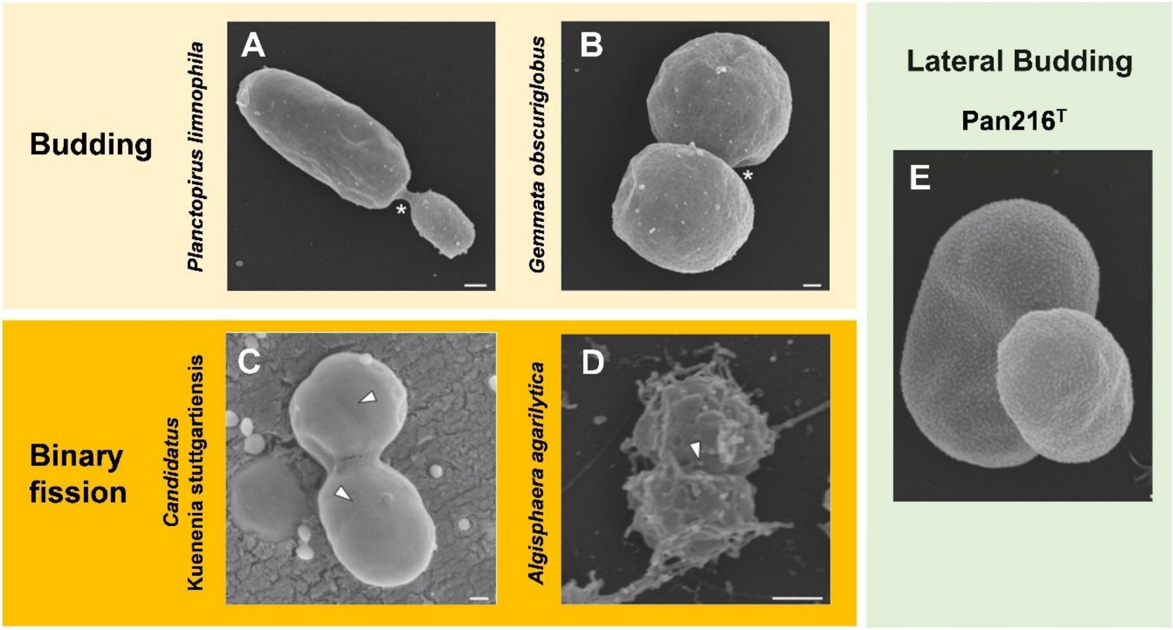

Figure S3. Cell division modes in the phylum *Planctomycetota*. *P. limnophila* (A) and *G. obscuriglobus* (B) divide by polar budding (asterisk), a common feature of members of the class *Planctomycetia*. In contrast, members of the classes *Candidatus* Brocadiae and *Phycisphaerae* divide by binary fission (C, D, white arrow head). Strain Pan216^T (E) displays an uncommon mode of cell division by forming daughter cells originating from the midcell (lateral budding). Scale bar 0.2 µm.

Supplementary Movies

Movie S1. Cell division of *Planctopirus limnophila*.

Movie S2. Cell division of *Gemmata obscuriglobus*.

Movie S3. Cell division of strain Pan216^{T}.

Supplementary Tables

Table S1. Operational taxonomic units (OTUs) obtained from surface seawater and the transition zone samples for the attached-living (2.7 µm) and free-living fraction (0.22 µm). (A) attached fraction surface water, (B) free-living fraction surface water, (C) attached fraction transition zone, (D) free-living fraction transition zone.

Table S2. Unique sequences belonging to members of the phylum *Planctomycetota* retrieved from the entire data set. The tentative assignment to described genera was performed manually based on the phylogenetic tree provided in Fig. S1.

Tables S1 and S2 are available as separate Excel files.

Table S3. Substrate utilization pattern of strain Pan216^T

Adonitol	+
Fermented rumen extract	+
Gluconate	+
Lactate	+
Maltose	+
Mannitol	+
Melizitose	+
N-acetylgalactosamine	+
N-acetylglucosamine	+
Ornithine	+
Protocatechuate	+
Raffinose	+
Shikimate	+
Sucrose	+
Trehalose	+
Xylose	+
Yeast extract	+
Cellobiose	(+)
Fructose	(+)
Glucose	(+)
Lactose	(+)
Mannose	(+)
Propionate	(+)
Rhamnose	(+)

Abbreviations: +: average growth above 150%, (+) average growth above 140%. The percent values refer to a culture without a supplemented carbon source. Tested substrates included Arabinose, Cellobiose, Erythrose, Erythrulose, Fructose, Fucose, Galactose, Glucose, Lactose, Lyxose, Maltose, Mannose, Melizitose, Raffinose, Rhamnose, Sorbose, Sucrose, Trehalose, Xylose, Glucosamine, *N*-acetyl-glucosamine, *N*-acetylgalactosamine, Acetoin, Adonitol, Arabitol, Dulcitol, Lyxitol, Mannitol, *myo*-Inositol, Sorbitol, Xylitol, Alanine, Arginine, Asparagine, Aspartate, Cysteine, Glutamate, Glutamine, Glycine, Histidine, Hydroxy-Proline, Isoleucine, Leucine, Lysine, Methionine, Ornithine, Phenylalanine, Proline, Threonine, Tryptophan, Tyrosine, Valine, Adipate, Acetate, Ascorbate, Benzoate, Trimethoxy-benzoate, Butyrate, α-Hydroxybutyrate, β-Hydroxybutyrate, γ-Hydroxybutyrate, Isobutyrate, Caproate, Caprylate, Citrate, Isocitrate, Crotonate, Formate, Fumarate, Gluconate, 2-Oxogluconate, Glucuronate, 2-Oxoglutarate, Glycolate, Glyoxylate, Heptanoic acid, Isovalerate, Laevulinate, Lactate, Malate, Maleic acid, Malonate, Nicotinic acid, Oxaloacetate, Propionate, Protocatechuate, Pyruvate, Shikimate, Succinate, Tartrate, 2-Oxovalerate, Butanol, 1,2-Butandiol, 2,3-Butandiol, Ethanol, Ethylene glycol, Glycerol, Methanol, Propanol, 1,2-Propandiol, Fermented rumen extract, Laminarin, Tween 80, Casamino acids, Casein hydrolysate, Peptone, Yeast extract, Starch, Cellulose, Chitin, and Avicel.

Table S4. Cellular fatty acid profiles of the novel strain Pan216^T. The data were obtained using M1H NAG ASW (pH 8, 7d, 28 °C). Values represent percentages of the total fatty acids identified with the standard methods of the Microbial Identification System (MIDI Inc.; version 6.1) (1). Major components ≥ 9% (bold).

**Fatty acid**	
**Saturated**	
**14:0**	**20.54**
15:0	0.9
**16:0**	**35.47**
18:0	1.22
**Unsaturated**	
**18:1 ω7c**	**31.2**
20:1 ω7c	0.99
**Summed feature**	
**3[a]**	**9.69**

[a] Determined as summed feature 3 (16:1 ω7c and/or 15 iso 2-OH) by the DSMZ service unit according to the MIDI System; these two lipids have the same retention time in the GC analysis, so are called together here since they cannot be discriminated. Reference (2) referred to this peak as 16:1 ω7c.

Table S5. Core genome analysis based on the comparison of strain Pan216^T with either non-budding-, budding- or all sequenced planctomycetes

The table is provided as separate excel sheet.

Table S6. Genes encoding proteins involved in cell division and peptidoglycan biosynthesis.

Locus tag	Gene name
Pan216_22570	*marZ*
Pan216_23080	*murB_2*
Pan216_40180	*ftsK*
Pan216_48590	*murB_1*
Pan216_48500	*murF*
Pan216_48550	*murC*
Pan216_48560	*murD*
Pan216_48570	*murE*
Pan216_48580	*murA*

Table S7. Putative S-layer proteins in strain Pan216.
The table is provided as separate excel sheet.

References

1. Damsté, JSS, Rijpstra, WIC, Hopmans, EC, Weijers, JW, Foesel, BU, Overmann, J, Dedysh, SN, 2011. 13,16-Dimethyl octacosanedioic acid (iso-diabolic acid), a common membrane-spanning lipid of *Acidobacteria* subdivisions 1 and 3. *Appl Environ Microbiol* 77: 4147-4154.
2. Sasser, M, Identification of bacteria by gas chromatography of cellular fatty acids. MIDI technical note 101. Newark, DE: MIDI Inc., 1990.
